# Assessing the Ecotoxicologic Hazards of a Pandemic Influenza Medical Response

**DOI:** 10.1289/ehp.1002757

**Published:** 2011-03-02

**Authors:** Andrew C. Singer, Vittoria Colizza, Heike Schmitt, Johanna Andrews, Duygu Balcan, Wei E. Huang, Virginie D.J. Keller, Alessandro Vespignani, Richard J. Williams

**Affiliations:** 1 Centre for Ecology and Hydrology, Wallingford, Oxfordshire, United Kingdom; 2 INSERM, U707, Paris, France; 3 UPMC Université Paris 06, Faculté de Médecine Pierre et Marie Curie, UMR S 707, Paris, France; 4 Computational Epidemiology Laboratory, Institute for Scientific Interchange, Turin, Italy; 5 Institute for Risk Assessment Sciences, Utrecht University, Utrecht, the Netherlands; 6 Department of Civil and Structural Engineering, University of Sheffield, Sheffield, United Kingdom; 7 Center for Complex Networks and Systems Research, School of Informatics and Computing, and; 8 Pervasive Technology Institute, Indiana University, Bloomington, Indiana, USA; 9 Institute for Scientific Interchange, Turin, Italy

**Keywords:** antibiotics, antiviral, bacterial pneumonia, ecotoxicity, epidemiologic modeling, influenza, pandemic, Tamiflu, wastewater treatment plant

## Abstract

Background: The global public health community has closely monitored the unfolding of the 2009 H1N1 influenza pandemic to best mitigate its impact on society. However, little attention has been given to the impact of this response on the environment. Antivirals and antibiotics prescribed to treat influenza are excreted into wastewater in a biologically active form, which presents a new and potentially significant ecotoxicologic challenge to microorganisms responsible for wastewater nutrient removal in wastewater treatment plants (WWTPs) and receiving rivers.

Objectives: We assessed the ecotoxicologic risks of a pandemic influenza medical response.

Methods: To evaluate this risk, we coupled a global spatially structured epidemic model that simulates the quantities of antivirals and antibiotics used during an influenza pandemic of varying severity and a water quality model applied to the Thames catchment to determine predicted environmental concentrations. An additional model was then used to assess the effects of antibiotics on microorganisms in WWTPs and rivers.

Results: Consistent with expectations, our model projected a mild pandemic to exhibit a negligible ecotoxicologic hazard. In a moderate and severe pandemic, we projected WWTP toxicity to vary between 0–14% and 5–32% potentially affected fraction (PAF), respectively, and river toxicity to vary between 0–14% and 0–30% PAF, respectively, where PAF is the fraction of microbial species predicted to be growth inhibited (lower and upper 95% reference range).

Conclusions: The current medical response to pandemic influenza might result in the discharge of insufficiently treated wastewater into receiving rivers, thereby increasing the risk of eutrophication and contamination of drinking water abstraction points. Widespread drugs in the environment could hasten the generation of drug resistance. Our results highlight the need for empirical data on the effects of antibiotics and antiviral medications on WWTPs and freshwater ecotoxicity.

During the course of a pandemic, large quantities of drugs are projected to be used to treat cases of influenza and influenza-associated complications (Lim 2007), mitigate the spread of the epidemic, and reduce the burden on the health care system [U.S. Centers for Disease Control and Prevention (CDC) 2010; World Health Organization 2009]. The drug use patterns associated with intervention measures necessarily vary depending on the transmission potential of the new virus, its pathogenicity, and the rate of occurrence of mild to severe illness and complications.

Under any pandemic scenario, there is potential for environmental impact because most ingested antivirals (used for prophylaxis as well as treatment) and antibiotics (used to treat secondary bacterial infections such as pneumonia) are excreted from the human body and released into the wastewater treatment system in a biologically active form (Singer et al. 2007, 2008). Antibiotic and antiviral use during an influenza pandemic can far exceed that of interpandemic use—particularly in the case of antivirals, which are infrequently used in the United Kingdom for seasonal influenza (Kramarz et al. 2009)—and, as a result, presents a new and potentially significant ecotoxicologic challenge to wastewater treatment plants (WWTPs) and receiving rivers (PREPARE Initiative 2009).

Antibiotic use, and its associated ecotoxicologic hazards, might be reduced by assuming a systematic use of antiviral drugs to mitigate the likelihood and severity of influenza infections and resulting complications, including bacterial pneumonia (Kaiser et al. 2003; Yu et al. 2010). However, the concomitant increase in antiviral use in an effort to reduce secondary bacterial infections could exacerbate any ecotoxicologic hazard associated with the antiviral. Disruption of microorganisms responsible for nutrient removal in WWTPs from the combined ecotoxicologic effects of antibiotic and antiviral exposure could result in insufficiently treated wastewater entering the receiving rivers, leading to eutrophication, loss of aquatic life, and fish kills. Similar environmental effects have been witnessed in areas after periods of brief but intense heavy rainfall, which forces raw sewage directly into receiving rivers (Even et al. 2004); this phenomenon is particular to areas employing combined sewage overflows, as is the case in much of the United Kingdom. These scenarios typify the risk induced by interdependencies among social systems, infrastructures, and the environment; the failure of a single entity or cluster of entities can cause a chain reaction, which can disrupt the entire system.

In this article we provide the first quantitative assessment of the potential environmental hazards associated with the medical response to a pandemic. We focus on the Thames catchment in England as a case study (Figure 1), because it is one of the most populous and production-dense river catchments in the world (Merrett 2007). To quantify the relevant environmental risk, we integrated a spatially structured global epidemic model with a water quality model and toxicity models to produce *ab initio* estimates of drug use patterns, estimates of their release into WWTPs, projected levels of contamination of the receiving rivers, and resulting microbial ecotoxicity.

**Figure 1:**
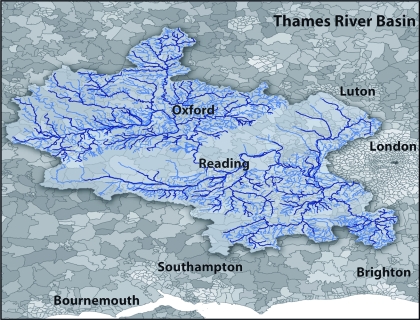
Illustration of the Thames River Basin boundary. Dark blue represents river stretches receiving WWTP effluent within the LF2000‑WQX; light blue represents river stretches upstream of the first WWTP found within the LF2000‑WQX. A river stretch is defined by the length of river bounded at both ends by an input to or abstraction from the river (e.g., another river, WWTP, drainage canal, abstraction point).

## Methods

*Epidemic simulations.* We used the Global Epidemic and Mobility (GLEaM) model (Balcan et al. 2009a) to generate *in silico* epidemics, simulating the numbers of influenza cases and secondary bacterial infection cases at each stage of disease progression and the quantities of antiviral drugs (used for prophylaxis and treatment) and antibiotics (used to treat secondary bacterial infections) used within each geographic census area, with projections down to the spatial resolution scale of 0.25° and a time resolution of 1 day. A detailed description of the model and model parameters is provided in Supplemental Material, Section 1 (doi:10.1289/ehp.1002757). In brief, the model mapped 6 billion individuals and integrated mobility data at the worldwide scale, including air travel and commuting patterns, to simulate the spread of infection among 3,362 geographic census area subpopulations defined around airports in 220 countries (Balcan et al. 2009a). The model simulates the evolution of influenza within each subpopulation, with each individual classified as susceptible, latent, infectious symptomatic, infectious asymptomatic, or permanently recovered/removed at each point in time (see Supplemental Material, Figure 2). The model accounts for seasonal effects through standard *a priori* assumptions on seasonal rescaling of influenza transmissibility (Balcan et al. 2009a; Colizza et al. 2007; Cooper et al. 2006) (see Supplemental Material, Table 1). The compartmentalization accounting for the development of influenza-associated complications (Balcan et al. 2009b) were based on the U.K. pandemic assumptions for complication, hospitalization, and intensive care unit admission rates (Balcan et al. 2009b; U.K. Department of Health 2009) (see Supplemental Material, Table 2). All epidemic simulations were initiated with a single symptomatic infectious individual and were allowed to evolve for 1 year. We considered for the analysis only simulations that resulted in a global outbreak, defined as the generation of new symptomatic cases in more than one country. Initial conditions assumed that the pandemic would start in Hanoi, Vietnam, on 1 October (Colizza et al. 2007). The integration of short- and long-range mobility infrastructures, and detailed demographic data with a seasonality scaling that impacts geographic areas differently, allowed for a fine-grained description of the epidemic.

**Figure 2:**
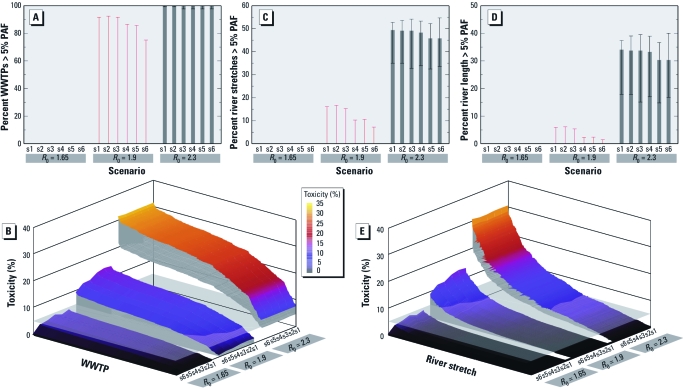
Predicted toxicity to microorganisms in WWTPs and river stretches resulting from exposure to antibiotics during influenza pandemics. Scenarios: s1, AVP = 0, rate of AVT = 30%, limited supply of Tamiflu; s2, 2 week AVP, AVP = 0.1%, rate of AVT = 30%, limited supply of Tamiflu; s3, 4 week AVP, AVP = 0.1%, rate of AVT = 30%, limited supply of Tamiflu; s4, 2 week AVP, AVP = 1%, rate of AVT = 30%, limited supply of Tamiflu; s5, 4 week AVP, AVP = 1%, rate of AVT = 30%, limited supply of Tamiflu; s6, AVP = 0, rate of AVT = 30%, unlimited supply of Tamiflu. (*A,C,D*) Percentage of WWTPs (*A*), river stretches (*C*), and river length (total length of the river stretches in the Thames River Basin; *D*) predicted to exceed the toxicity threshold of 5% PAF by transmission scenario (mild, moderate, and severe). Values shown are median and 95% RRs obtained from the drug use pattern predicted by the 1,000 stochastic runs of the GLEaM model. No bar is visible when the median value equals zero; this is the case, for example, for the mild and moderate scenarios. Note that antiviral treatment is assumed in the moderate and severe pandemic scenarios only, with 30% case detection and drug administration. Intervention with antivirals is modeled by assuming that each country has limited stockpiles of the drug [s1–s5; see Supplemental Material, Figure 4 (doi:10.1289/ehp.1002757)] (Colizza et al. 2007; Singer et al. 2008) or that each country can count on a hypothetical unlimited supply of drugs (s6). PAF calculations are based on the antibiotic sensitivity distributions of human pathogens and a combination of two mixture toxicity models. (*B* and *E*) Absolute toxicity, shown as a percentage of microbial species predicted to be growth inhibited (PAF) per each WWTP (*B*) and river stretch (*E*) according to the pharmaceutical mitigating conditions explored, in the mild, moderate, and severe transmission scenarios.

**Table 1:** Projected concentrations of antibiotics and Tamiflu in the Thames River Basin.

Antibiotics (μg/L)			Tamiflu (μg/L)		
Scenario	Mean ± SD	Maximum	Mean ± SD	Maximum
*R*_0_ = 1.65								
s1		0.085 ± 0.088		0.476		0.186 ± 0.192		1.04
s2		0.082 ± 0.084		0.445		1.12 ± 1.15		6.09
s3		0.083 ± 0.084		0.447		1.20 ± 1.21		6.47
s4		0.073 ± 0.074		0.400		11.1 ± 11.2		60.8
s5		0.070 ± 0.072		0.384		11.1 ± 11.3		60.6
s6		0.013 ± 0.014		0.073		0.027 ± 0.027		0.149
*R*_0_ = 1.9								
s1		0.741 ± 0.744		3.95		1.00 ± 1.00		5.31
s2		0.690 ± 0.706		3.77		1.16 ± 1.19		6.33
s3		0.719 ± 0.731		3.90		1.47 ± 1.49		7.96
s4		0.552 ± 0.563		3.01		11.0 ± 11.2		60.0
s5		0.418 ± 0.427		2.27		11.5 ± 11.7		62.4
s6		0.294 ± 0.298		1.59		0.37 ± 0.38		2.02
*R*_0_ = 2.1								
s1		14.8 ± 15.0		80.5		21.3 ± 21.3		102
s2		14.5 ± 14.8		80.6		21.0 ± 21.3		103
s3		14.5 ± 14.8		79.9		21.1 ± 21.2		102
s4		14.0 ± 14.2		75.9		20.7 ± 20.8		99.1
s5		13.1 ± 13.3		69.3		19.6 ± 19.9		103
s6		13.2 ± 13.4		72.3		19.6 ± 20.0		108
Scenarios: s1, AVP = 0, rate of AVT = 30%, limited supply of Tamiflu (i.e., based on the available stockpiles of each country) [see Supplemental Material, Figure 4 (doi:10.1289/ehp.1002757)]; s2, 2 weeks of AVP, AVP = 0.1%, rate of AVT = 30%, limited supply of Tamiflu; s3, 4 weeks of AVP, AVP = 0.1%, rate of AVT = 30%, limited supply of Tamiflu; s4, 2 weeks of AVP, AVP = 1%, rate of AVT = 30%, limited supply of Tamiflu; s5, 4 weeks of AVP, AVP = 1%, rate of AVT = 30%, limited supply of Tamiflu; s6, AVP = 0, rate of AVT = 30%, unlimited supply of Tamiflu, (i.e., assuming that each country can count on unlimited stockpiles of Tamiflu). Mean values are inclusive of all excreted antibiotics. Values reflect the median epidemic scenario for each condition and the mean concentration for all 461 river stretches used within the LF2000‑WQX model.								

Given the large uncertainties associated with an emerging influenza pandemic, we explored different scenarios, ranging from mild transmission potential with a basic reproductive number (*R*_0_) of 1.65, to a moderate situation where *R*_0_ = 1.9, to a severe scenario where *R*_0_ = 2.3. *R*_0_ indicates the average number of infections generated by an infectious individual in a fully susceptible population (Anderson and May 1991). These *R*_0_ values, which are consistent with recent estimates of the current H1N1 pandemic (CDC 2010; Fraser et al. 2009; Yang et al. 2009) up to available estimates for the 1918 pandemic (Mills et al. 2004), correspond to the nominal values of *R*_0_ and, as such, do not reflect the seasonal influence on *R*_0_, which is accounted for within the GLEaM model (Colizza et al. 2007; Cooper et al. 2006).

In addition to analyzing ecotoxicologic hazards corresponding to differing levels of pandemic severity, we also considered different pharmaceutical mitigation strategies that we assumed differed according to the selected transmission scenarios. In the case of a mild pandemic (*R*_0_ = 1.65), we assumed no large-scale antiviral treatment (AVT), consistent with the response measures implemented during the 2009 H1N1 pandemic (CDC 2010; U.K. Department of Health 2009; World Health Organization 2009). For moderate and severe pandemic scenarios, we assumed the implementation of AVT with Tamiflu (F. Hoffmann-La Roche Ltd, Basel, Switzerland) in all countries with available stockpiles [see Supplemental Material, Figure 4 (doi:10.1289/ehp.1002757)] (Colizza et al. 2007; Singer et al. 2008). This mitigation strategy was modeled assuming a conservative 30% successful case detection and antiviral treatment AVT rate (Balcan et al. 2009c; Colizza et al. 2007; Flusurvey.org.uk 2010). We assumed AVT resulted in a 1-day reduction of the infectious period, a reduced transmissibility of the infection, and a reduced complication rate (see Supplemental Material, Section 1 and Table 1) (Balcan et al. 2009b; Ferguson et al. 2006; Germann et al. 2006). We also evaluated the effects of antiviral prophylaxis (AVP) provided to 0.1% or 1% of the population for 2 or 4 weeks, respectively, from the start of the outbreak (Ferguson et al. 2006; Germann et al. 2006; Health Protection Agency 2009). The case with no prophylaxis was also considered. We based our assumptions regarding antibiotic treatment for influenza-associated complications on the empirical guidelines of the British Infection Society, British Thoracic Society, and Health Protection Agency (Lim 2007) (see Supplemental Material, Table 3). Full details and sensitivity analysis on these parameters are reported in Supplemental Material, Section 1.

*Environmental fate and ecotoxicity analysis.* The coupling of the GLEaM model with a point-source water quality model, Low Flows 2000—Water Quality Extension (LF2000-WQX) (Rowney et al. 2009), allowed for an accurate description of the evolution of the pandemic and the environmental release of antiviral medications and antibiotics. First, we estimated drug excretion into WWTPs based on the GLEaM model and pharmacologic data concerning the percentage of each drug released in the feces and urine as the parent chemical or as a biologically active metabolite [for details, see Supplemental Material, Section 2 and Table 4 (doi:10.1289/ehp.1002757)]. We used LF2000-WQX to estimate spatially explicit statistical distributions of river concentrations of antivirals and antibiotics discharged from WWTPs after accounting for dilution and dissipation processes in the river (see Supplemental Material, Section 3). The LF2000-WQX software (Keller and Young 2004; Williams et al. 2009) is a geographic information-based system that uses a Monte Carlo mixing-model approach to combine statistical estimates of chemical loads at specific emission points (e.g., WWTPs) with estimated river flow duration curves to generate spatially explicit statistical distributions of chemicals for the whole river network. For this analysis, we assumed that the pharmaceutical load in WWTP influent per person per day constant and fixed at the mean peak value for the pandemic. In addition, we assumed that WWTPs were the only sources of drugs, that drugs were not removed in the WWTP or degraded in the water column, and that background concentrations in the river stretches and lateral inflows were zero.

Assumptions and sensitivity analysis for the analysis of ecotoxicity are described in detail in Supplemental Material, Section 4 (doi:10.1289/ehp.1002757). In brief, we focused our ecotoxicologic analysis on eight antibiotics (amoxicillin, cefotaxime, cefuroxime, clarithromycin, doxycycline, erythromycin, levofloxacin, and moxifloxacin), because Tamiflu itself has not been shown to exhibit acute toxicity (Accinelli et al. 2010; Bartels and von Tümpling 2008; Hutchinson et al. 2009; Kelleher and Dempsey 2007; Saccà et al. 2009; Straub 2009). The ecotoxicologic hazard posed by each scenario and its respective antibiotic use pattern was measured in terms of the “potentially affected fraction” (PAF) of microbial species within a WWTP or a river, which was projected to be growth inhibited by antibiotics exposure. The PAF was calculated by use of bacterial species sensitivity distributions of antibiotic toxicity constructed from compilations of minimum inhibitory concentrations (MICs; see Supplemental Material, Figure 8). Because antibiotic sensitivity data for microorganisms in WWTPs (i.e., the microorganisms responsible for the removal of nutrients from wastewater before discharge into receiving rivers) are limited, we based effect assessments on MICs of predominantly clinically relevant microorganisms from the European Committee on Antimicrobial Susceptibility Testing (EUCAST) breakpoint database (EUCAST 2009). The database includes breakpoints from resistance surveillance programs, published articles, the pharmaceutical industry, veterinary programs, and individual laboratories. We accounted for the presence of multiple antibiotics through mixture toxicity models (De Zwart and Posthuma 2005). A PAF of 5% was used as a pragmatic threshold to define the maximum fraction of species present in a community that could be inhibited without any anticipated loss of function to the “system” (European Chemicals Agency 2008).

*Effects of Tamiflu on bacterial biofilms.* Soong et al. (2006) demonstrated the efficiency of oseltamivir carboxylate (OC; the active metabolite of Tamiflu) to inhibit biofilm formation of the pathogen *Pseudomonas aeruginosa* (Soong et al. 2006). The extent to which Tamiflu and OC will inhibit biofilm formation in environmentally relevant strains of microorganisms in WWTPs is unknown, but such inhibition could interfere with the nutrient-removing microorganisms within WWTPs and thereby contribute to WWTP failure and contamination of receiving rivers and downstream drinking water. Moreover, there is a risk that the effects might be further exaggerated when combined with a high load of antibiotics, as we projected in this study. Therefore, we used a cell attachment assay to determine the influence of Tamiflu exposure on biofilm formation of environmentally relevant bacterial strains (Djordjevic et al. 2002; O’Toole and Kolter 1998), as described in detail in Supplemental Material, Section 5 (doi:10.1289/ehp.1002757). In brief, we exposed nine environmental microorganisms and one clinical microorganism (see Supplemental Material, Table 7) to two concentrations of OC [28.4 and 284 mM (0.1 and 1.0 mg/L, respectively)] and observed the relative differences in the extent of biofilm formation in a 96-well plate format.

## Results

For each pandemic scenario, we first estimated the quantity of antibiotics and antivirals reaching WWTPs at the peak of the pandemic. In the case of a mild pandemic (*R*_0_ = 1.65), we projected antibiotic use to increase by a negligible 1% over interpandemic use of the same antibiotics reported for England in 2007–2008 [95% reference range (RR), 0.4–23%; for estimated background antibiotic use during interpandemic periods, see Supplemental Material, Table 5 (doi:10.1289/ehp.1002757)]. We obtained the RR from the RR of the drug use pattern predicted by the GLEaM model. Antibiotic use was projected to increase by 13% (95% RR, 1–83%) and 252% (95% RR, 158–279%), respectively, over interpandemic use for moderate and severe transmission scenarios with 30% of cases receiving AVT intervention.

**Figure 3:**
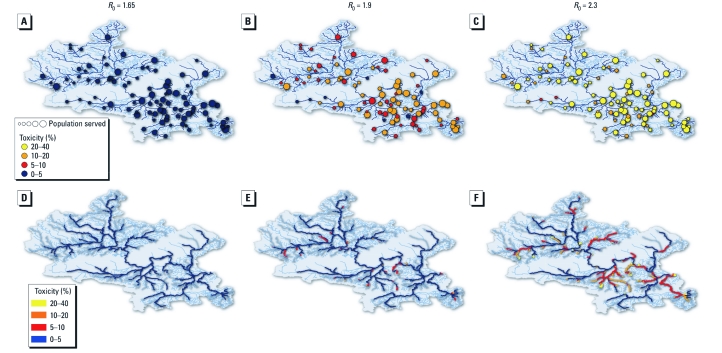
Maps showing the predicted toxicity of wastewater in WWTPs (*A–C*) and river stretches (*D–F*) corresponding to the drug use patterns shown in Figure 1C,D and Supplemental Material, Figure 3 (doi:10.1289/ehp.1002757), respectively, assuming no AVP. Toxicity values are binned and color coded as in Figure 2B and E. In *A*, individual WWTPs are indicated by circles that are scaled to indicate the size of the population served by each plant.

Projected microbial ecotoxicity for each WWTP for the different transmission scenarios and pharmaceutical interventions are shown in Figures 2B and 3A–C. The entire RR of toxicity in the mild pandemic is below the toxicity threshold of 5% PAF for all WWTPs. In a moderate pandemic, projected WWTP toxicity varied between 0–3% and 0–14% PAF for the least and most affected WWTPs, respectively (ranges represent the lower and upper 95% RRs). In the moderate pandemic scenario (*R*_0_ = 1.9), we projected the median number of WWTPs in the Thames River Basin with a PAF > 5% to be 0, but the upper 95% bound of the RR was > 74%, reflecting a realistic worst-case scenario (Figure 2A). In a severe pandemic, projected WWTP toxicity varied between 5–9% and 22–32% PAF for the least and most affected WWTPs, respectively. A PAF > 5% was projected in nearly all of the WWTPs when the lower bound of the 95% RR was considered in a severe pandemic.

A mild and moderate pandemic are unlikely to pose a significant ecotoxicologic hazard in the Thames basin, as shown in Figures 2C,D and 3D–F. Mean concentrations of total antibiotics in the Thames catchment were projected to be < 0.09 and < 0.8 μg/L for a mild and moderate pandemic, respectively (Table 1). In a moderate pandemic, projected river toxicity varied between 0 to < 1% and 0–14% PAF (ranges represent lower and upper 95% RR) for the least and most affected river stretches, respectively (Figure 2C,E). [A river stretch is defined by the length of river bounded at both ends by an input to or abstraction from the river (e.g., another river, WWTP, drainage canal, abstraction point).] In a severe pandemic, projected river toxicity varied between 0 to ≤ 1% and 21–30% PAF for the least and most affected river stretches, respectively. We projected a severe pandemic to average < 15 μg/L for the sum of antibiotics and result in a maximum environmental concentration of 80 μg/L. These concentrations would exceed the 5% PAF threshold in about half the river stretches under all prophylaxis interventions considering the upper bound of the 95% RR (lower bound > 30%), equating to approximately 35–40% of the total river length within the basin (lower bound, 15–18%; Figure 3F). [River length was evaluated on the total length of the river stretches in the Thames River Basin.]

The same WWTPs or rivers generated the highest ecotoxicity risk across the set of interventions explored for any one transmission scenario (Figure 2B,E). The WWTPs at higher ecotoxicologic risk are often ones serving larger populations (Figure 3A–C). Both WWTPs and river stretches reaching the highest toxicity values tended to be located closer to London, where most drinking water abstraction points are located for the London area (Rowney et al. 2009). Hence, the increased risk of drinking water contamination might result in the need for additional water treatment measures.

Interpandemic use of Tamiflu in the United Kingdom has been reported to be negligible (Kramarz et al. 2009), implying that any substantial increase will result from the pandemic. We projected a mild and moderate pandemic with AVP > 0, to generate mean concentrations of OC (the active form of the prodrug Tamiflu) between 1.1 and 11.5 μg/L in the Thames catchment (Table 1). A more severe pandemic, regardless of AVP, was projected to result in mean concentrations of OC > 100 μg/L, which is consistent with previous projections of a severe pandemic in southern England (Singer et al. 2007).

In the biofilm formation assay, we observed statistically significant (*p* < 0.05) declines in biofilm formation relative to control in 20% and 10% of the cases when bacteria were exposed to 0.1 mg/L and 1 mg/L OC, respectively [see Supplemental Material, Section 5 and Figure 12 (doi:10.1289/ehp.1002757)].

## Discussion

To our knowledge, this is the most comprehensive study to date to estimate environmental concentrations of pharmaceuticals, at a catchment scale, for an influenza pandemic, because it includes pandemics of varying severities and mitigation strategies. This is the first study to identify the ecotoxicologic hazard to WWTPs from antibiotic use during an influenza pandemic, as well as the first to attempt to quantify the nature of hazard posed by the antiviral medication Tamiflu to microbial biofilm formation.

High concentrations of neuraminidase inhibitor antivirals, such as Tamiflu, zanamivir, and peramivir, might result in acute ecotoxicity during an influenza pandemic, although existing literature suggests little cause for concern for typical freshwater and marine ecotoxicology assay organisms (Accinelli et al. 2010; Escher et al. 2010; Hutchinson et al. 2009; Singer et al. 2008; Straub 2009). However, there is growing evidence to suggest that high concentrations of neuraminidase inhibitors in WWTPs and the environment might increase the risk of disrupting microbial biofilms, which has implications for WWTP floc and biofilm stability and the removal of nutrients from wastewater before discharge into receiving rivers (Singer et al. 2008). Slater et al. (2011) simulated influenza-pandemic dosing of antibiotics and antivirals for 8 weeks in an aerobic granular sludge sequencing batch reactor, operated for enhanced biological phosphorus removal (EBPR). They found evidence of changes to bacterial community structure and disruption to EBPR and nitrification during and after high-OC dosing. That study indicated the risk of destabilizing microbial consortia in WWTPs as a result of high concentrations of bioactive pharmaceuticals during an influenza pandemic.

Parker et al. (2009) showed that OC significantly inhibits *Streptococcus pneumoniae* NanA sialidase as reflected in decreased biofilm formation, with an IC_50_ (concentration necessary to inhibit enzyme activity by 50%) of 2 mM (568 mg/L) and an IC_30_ of 28.4 mg/L. They reported an IC_20_ of 10 μM OC (2.84 mg/L), which reflects the lowest tested concentration, but the decline in biofilm formation was not statistically significant (*p* > 0.05) (Parker et al. 2009). Soong et al. (2006) demonstrated similar levels of inhibition in *Psueodomonas aeruginosa* NanP sialidase with an approximate IC_50_ of 2.5 mg/L, nearly identical to that of NanP. Notably, Soong et al. (2006) demonstrated that peramivir had an IC_50_ of approximately 25 μM (8.2 mg/L) and an IC_30_ at a much lower, more environmentally relevant concentration of 0.025 μM (8.2 μg/L).

In a preliminary assay, we exposed nine environmental microorganisms and one clinical microorganism to two concentrations of Tamiflu and observed statistically significant (*p* < 0.05) declines in biofilm formation. Although we assayed the parent compound Tamiflu rather than its active antiviral metabolite (OC), 20% of the parent chemical reached the environment and thus has environmental significance in terms of biofilm exposure. The mechanism of biofilm inhibition by neuraminidase inhibitors remains undetermined.

We argue that there will be increasing risk during an influenza pandemic of antibiotic-mediated disruption of WWTP microorganisms. Increased antibiotic exposure could compromise vital and obligate microbial functions in WWTPs such as ammonium oxidation and nitrogen and phosphorus removal (Alighardashi et al. 2009; Louvet et al. 2010a, 2010b; Singer et al. 2008). Because pandemic influenza is likely to peak in winter months, the highest drug load will pass through WWTPs at their least effective time period, thereby maximizing the likelihood of WWTPs breaching compliance on discharged wastewater (PREPARE Initiative 2009).

Notably, we based the hazards identified in this study on MIC values of clinically relevant microorganisms, because experimental data on antibiotic toxicity in WWTPs are too scarce to be used for effects modeling. Potentially, the toxicity of antibiotics to WWTP microorganisms might differ from their effects on the clinically relevant microorganisms within the EUCAST database. Also, the organization of bacteria into biofilms will undoubtedly influence antibiotic toxicity *in situ*. However, recent data on erythromycin toxicity obtained in activated sewage sludge batch reactors show that MIC-based predictions agree with test results of an antibiotic “shock” (Louvet et al. 2010b). Within the variability of the experimental data, projected toxicity matched experimentally determined effects [see Supplemental Material, Section 4.5 and Table 6 (doi:10.1289/ehp.1002757)]. We therefore argue that it is defendable to use MIC values as a first approximation of antibiotic effects and at the same time stress the need for verification of possible effects in experimental studies. Should the WWTP microorganisms survive the initial toxic effects of the bolus of pharmaceuticals at the outset of a pandemic, increased biofilm thickness, changes in community composition, and horizontal transfer of antibiotic resistance might contribute to preserving WWTP function during a pandemic.

Sublethal levels of antibiotics have been shown to promote the development of antibiotic resistance in bacteria. Each potential extrinsic source of resistance genes, either in the environment or among commensal organisms, increases the chance of acquired resistance in a pathogen (Knapp et al. 2009), thereby potentially hastening the appearance of antibiotic resistant bacteria in humans (Smith et al. 2002). Hence, increased antibiotic use and release into the environment during an influenza pandemic might increase the environmental reservoir of antibiotic resistance, which has short- and long-term public health implications. Similarly, the release of active antivirals into rivers might hasten the generation of antiviral-resistant viruses in influenza-infected wildfowl, as previously discussed (Singer et al. 2007, 2008; Söderström et al. 2009).

Intervention strategies for the mitigation of a pandemic can strongly vary across countries, depending on policies and outbreaks experienced. In the moderate and severe transmission scenarios, we assumed that AVT was administered to 30% of influenza cases and considered varying values of the rate of complications. Larger administration rates of antiviral drugs for treatment may further reduce the incidence of secondary infections during the pandemic, thus reducing antibiotic use and its associated environmental risk, but at the cost of increasing the ecotoxicologic effects from additional antiviral use. A reduction in antibiotic use might alternatively be achieved through the use of a prepandemic/universal influenza vaccine (Leroux-Roels and Leroux-Roels 2009), as well as a multivalent pneumonia vaccination campaign (CDC 2009; Chien et al. 2010; U.K. Department of Health 2007).

## Conclusions

Widespread WWTP failures were not reported during the current H1N1 pandemic, as was projected by this study for a mild transmission scenario. However, future pandemics, depending on their severity, might test the resilience of WWTPs because of increased pharmaceutical use. Even a relatively small decline in the ability of WWTP microorganisms to remove wastewater nutrients (< 10%) could result in a significant pollution event when compounded for all WWTPs within a river. The projected ecotoxic effects of antivirals and antibiotics on WWTP biofilms could be considerable at the peak of a moderate or severe pandemic. Our current knowledge base is inadequate to rule out the potential for disruptions to wastewater treatment, widespread river pollution, degradation of drinking water quality, and the spread of antiviral and antibiotic resistance. The global nature of the GLEaM model and the availability of other regional catchment models enable the application of this study to other conditions and catchments. The data on the 2009 pandemic should be seen as a window of opportunity to gain further insight into the unique risks posed by a robust pharmaceutical response to influenza pandemics as it relates to the local environment, climate, and demographics.

## Supplemental Material

(1.1 MB) PDF
