# Supplementary material for: Assessing the Ecotoxicologic Hazards of a Pandemic Influenza Medical Response
Source: Environ Health Perspect. 2011 Mar 2;119(8):1084–90. doi: 10.1289/ehp.1002757 (PMC3237342; doi:10.1289/ehp.1002757)
Supplement: (1.1 MB) PDF [file ehp.1002757.s001.pdf]

Supplemental Material

Assessing the Ecotoxicologic Hazards of a Pandemic Influenza Medical Response

Andrew C. Singer*[1], Vittoria Colizza[2,3,4], Heike Schmitt[5], Johanna Andrews[6], Duygu Balcan[7,8], Wei E. Huang[6], Virginie D. J. Keller[1], Alessandro Vespignani[7,8,9], Richard J. Williams[1]

[1] Centre for Ecology & Hydrology, Wallingford, Oxfordshire, U.K
[2] INSERM, U707, Paris F-75012, France
[3] UPMC Université Paris 06, Faculté de Médecine Pierre et Marie Curie, UMR S 707, Paris F75012, France
[4] Computational Epidemiology Laboratory, Institute for Scientific Interchange, Turin, Italy
[5] Institute for Risk Assessment Sciences, Utrecht University, Utrecht, The Netherlands
[6] Department of Civil and Structural Engineering, University of Sheffield, Sheffield, UK
[7] Center for Complex Networks and Systems Research, School of Informatics and Computing, Indiana University, Bloomington, IN, USA
[8] Pervasive Technology Institute, Indiana University, Bloomington, IN, USA
[9] Institute for Scientific Interchange, Turin, Italy

Table of Contents

Section	Section Heading	Page
1.0	Global Epidemic and Mobility Modeler	3
1.1	Epidemic dynamic model: pandemic influenza and influenza-associated pneumonia	7
1.2	Pharmaceutical model	14
1.3	Scenarios	15
2.0	Fate of Pharmaceuticals in the Environment	16
2.1	Excretion and Dilution of Pharmaceuticals in WWTP	16
2.2	Low Flows 2000—Water Quality Extension	21
2.3	Background level of pharmaceuticals in the Thames River	22
3.0	Integration of GLEAM with LF2000-WQX	24
4.0	Toxicity assessment	25
4.1	Establishment of species sensitivity distributions	26
4.2	Calculation of the combined effects of the antibiotic mixture	28
4.3	Impact of different toxicity measure	31
4.4	Toxicity of background levels of antibiotics	31
4.5	Uncertainties in characterizing toxicity	33
4.6	Sensitivity analysis: Rate of complications development	36
5.0	Bacterial growth and biofilms formation assays	39
5.1	Bacterial biofilms	39
5.2	Biofilm inhibition assays	40
6.0	References	42

Tables

1. Parameter description and values 6
2. Severity assessment used to determine the management of influenza-related pneumonia in patients admitted to hospital (CURB-65 score), and corresponding compartments 12
3. Preferred and alternative empirical antibiotic treatment regimens for pneumonic influenza-associated complications 16
4. Percentage of parent pharmaceuticals investigated in this study excreted in the faeces and urine unchanged and/or as a bioactive metabolite 18
5. Estimated concentration of antibiotics in English WWTP during an inter-pandemic period 23
6. Comparison of predicted effects of erythromycin (PAF) with experimentally derived toxicity data 35
7. Bacterial strains selected for the biofilm bioassay 40

Figures

1. Schematic illustration of the stochastic metapopulation model. 4
2. Diagram flow of the transmission model 11
3. Predicted usage pattern of antiviral drugs and antibiotics per day in the three transmission scenarios investigated. 13
4. Tamiflu and Relenza stockpiles in national pandemic plans expressed in % population coverage 14
5. Annual mean wastewater produced per head per day (L) in the Thames Catchment as per WWTP database in LF2000-WQX 17
6. International perspective on litres of wastewater production head^{-1} day^{-1} 18
7. Percent removal of a range of antibiotics in WWTP from biodegradation 19
8. Species sensitivity distributions for 8 antibiotics based on MICs acquired from EUCAST database 28
9. Assessment of different toxicity model assumptions as a function of wastewater treatment plants 31
10. Predicted toxicity in WWTPs 37
11. Predicted toxicity in river stretches 38
12. Impact of Tamiflu exposure on microbial biofilm formation 42

1.0 GLobal Epidemic and Mobility modeler

The GLobal Epidemic and Mobility (GLEaM) computational modeler is a georeferenced metapopulation model based on three data/model layers as previously discussed (Balcan et al. 2009a; Balcan et al. 2009c). The first layer is based on the high resolution population database of the Gridded Population of the World project (Center for International Earth Science Information Network (CIESIN) Columbia University et al.) of the SocioEconomic Data and Applications Center of the Columbia University that estimates the population with a granularity given by a lattice of cells covering the whole planet at a resolution of 15×15 minutes of arc. The second layer refers to a human mobility model defined by the transportation and commuting networks characterizing the interactions and exchanges of individuals across subpopulations. The transportation mobility layer integrates air travel mobility obtained from the International Air Transport Association database (International Air Transport Association) that contains the list of worldwide airport pairs connected by direct flights and the number of available seats on any given connection. The combination of the population and mobility layers allows the subdivision of the world into georeferenced census areas defined with a Voronoi tessellation procedure around transportation hubs (Barrat et al. 2004; Colizza et al. 2007; Colizza et al. 2006a; Colizza et al. 2006b). These census areas define the subpopulations of the metapopulation model (Supplemental Figure 1). In particular, we identify 3,362 subpopulations centered around IATA airports in 220 different countries (Balcan et al. 2009c; Colizza et al. 2007). GLEaM integrates short scale mobility between adjacent subpopulations by considering commuting patterns worldwide as obtained from the data collected and analyzed from more than 29 countries in 5 continents across the world (Balcan et al. 2009a; Balcan et al. 2009c). The third layer is the epidemic dynamic model that defines the evolution of the infectious disease inside each subpopulation.

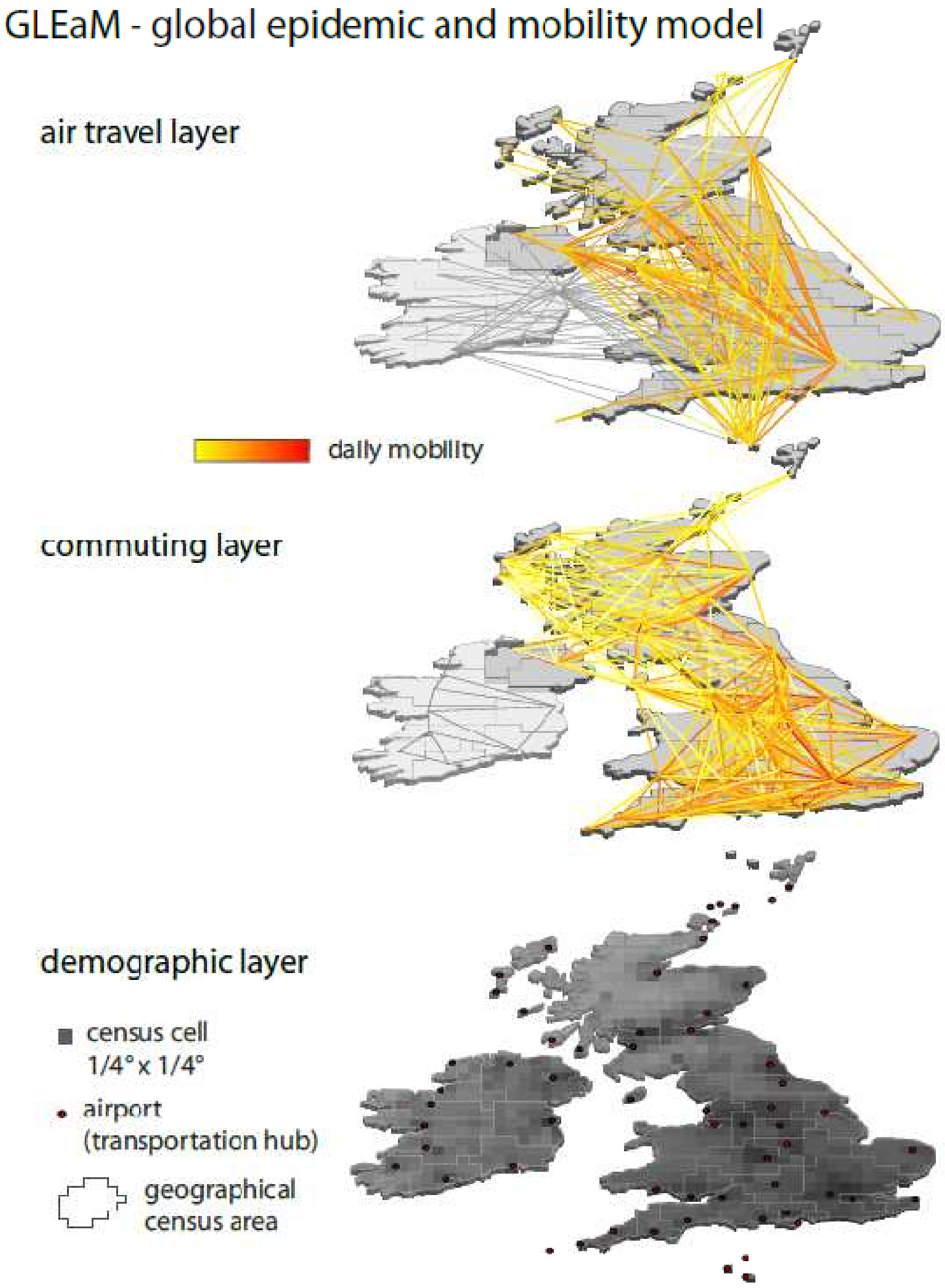

Supplementary Figure 1. Schematic illustration of the stochastic metapopulation model. GLEaM integrates high resolution demographic and mobility data defining geographical census areas connected by commuting patterns and air travel flows. The same resolution is used worldwide.

The model simulates the mobility of individuals from one subpopulation to another by a stochastic procedure in which the number of passengers of each compartment traveling from a subpopulation *j* to a subpopulation *l* is an integer random variable defined by the actual data from the airline transportation database. Short range commuting between subpopulations is modeled (Balcan et al. 2009a; Balcan et al. 2009c) with a time scale separation approach that defines the effective force of infections in connected subpopulations. The baseline infection dynamics takes place within each subpopulation and assumes the classic influenza-like illness compartmentalization in which each individual is classified by a discrete state such as susceptible, latent, infectious symptomatic, infectious asymptomatic or permanently recovered/removed. The model therefore assumes that the latent period is equivalent to the incubation period and that no secondary transmissions occur during the incubation period (Supplemental Figure 2). The following section will provide a detailed description of the compartmentalization. All transitions are modeled through binomial and multinomial processes to preserve the discrete and stochastic nature of the processes. The model generates *in silico* epidemics for which we can gather information such as prevalence and number of secondary cases for each subpopulation and with a time resolution of 1 day.

Supplemental Table 1. Parameter description and values.

Parameter	Value	Description
β		transmission rate
**AVP_L**	0, 10^{-3}, 10^{-2}	daily rate of AV distribution for prophylaxis of duration L
**L**	2w, 4w	duration of AV prophylaxis intervention; w=week
χ	$(10\ d)^{-1}$	inverse duration of 1 course of AV prophylaxis; d=day
ε	$(1.9\ d)^{-1}$	inverse duration of average latency period
μ	$(3\ d)^{-1}$	inverse duration of average infectious period
r_β	50%	relative infectiousness of asymptomatic infectious individuals
r_{AVTd}	$(2\text{-}AVE_I)/2$	relative infectiousness of infectious individuals under treatment
AVE_S	0.30	AV efficacy for susceptibility
AVE_D	0.60	AV efficacy for symptomatic disease
AVE_I	0.62	AV efficacy for infectiousness
p_a	33%	probability of being asymptomatic
p_t	50%	probability of traveling
p_P	15% [2%-40%]	complication rate
$p_{P\text{-}AV}$	αp_P	complication rate when the influenza case is treated with antivirals
α	54%	reduction factor for the probability of developing pneumonia following influenza treated with antivirals
p_{AVT}	0.3	probability of receiving AV drugs (includes case detection and prompt delivery/administration within 1day from symptoms onset)
p_{II}	11%	probability of pneumonia worsening from CURB-65=0,1 to CURB-65=2
p_{III}	1.65%	probability of pneumonia worsening from CURB-65=2 to CURB-65=3,4,5
μ_{AV}	$(\mu^{-1}-1)^{-1}$	inverse duration of average infectious period during therapeutic treatment
λ	$(5\ d)^{-1}$	inverse duration of CURB-65=0,1, CURB-65=2 and CURB-65=3,4,5

1.1 Epidemic dynamic model: pandemic influenza and influenza-associated pneumonia

Each geographical census area corresponds to a subpopulation in the metapopulation model, inside which we consider a Susceptible-Latent-Infectious-Recovered (SLIR) compartmental scheme, typical of influenza-like illnesses (ILIs), where each individual has a discrete disease state assigned at each moment in time. The contagion process, i.e., generation of new infections, is the only transition mechanism which is altered by short-range mobility, whereas all the other transitions between compartments are spontaneous and remain unaffected by the commuting (Balcan et al. 2009a; Balcan et al. 2009c). The rate at which a susceptible individual in subpopulation *j* acquires the infection, the so called force of infection λ_j, is determined by interactions with infectious persons either in the home subpopulation *j* or in its neighboring subpopulations on the commuting network. In the absence of intervention, each person in the susceptible compartment S_j contracts the infection with probability $\lambda_j \Delta t$ and enters the latent compartment L_j, where Δt is the time interval considered. Latent individuals exit the compartment with probability $\varepsilon \Delta t$, and transit to asymptomatic infectious compartment I_j^a with probability p_a or become symptomatic infectious with the complementary probability 1-p_a. Infectious persons with symptoms are further divided between those who can travel I_j^t, probability p_t, and those who are travel-restricted I_j^{nt}, with probability 1- p_t. All the infectious persons permanently recover with probability $\mu \Delta t$, entering the recovered compartment R_j in the next time step. Consult Supplemental Figure 1 for the compartmental model adopted and Supplemental Table 1 for the parameter values used.

In each subpopulation the variation of the number of individuals in each compartment [m] can be written at any given time step as

$$X_j^{[m]}(t + \Delta t) - X_j^{[m]}(t) = \Delta X_j^{[m]} + \Omega_j([m])$$

where the term $\Delta X_j^{[m]}$ represents the change due to the compartment transitions induced by the disease dynamics. The transport operator, $\Omega_j([m])$, represents the variations due to the traveling and mobility of individuals. The latter operator takes into account the long-range airline mobility and defines the minimal time scale of integration to 1 day. The mobility due to the commuting flows is taken into account by defining effective force of infections by using time scale separation approximations as previously discussed (Balcan et al. 2009a).

Antiviral prophylaxis and treatment. We model pharmaceutical interventions aimed at mitigating the impact and the spread of pandemic influenza on the population. We consider the use of antiviral drugs both for prophylaxis (AVP) and therapeutic treatment (AVT). The efficacy of the treatment is constrained by the administration of the drugs within the first 2 days from symptoms onset. It aims at: *(i)* reducing the probability of transmission of an infectious individual under treatment; *(ii)* reducing by 1 day the average duration of the infection. The use of AV drugs for prophylaxis aims at: *(i)* reducing the susceptibility to infection; *(ii)* reducing the infectiousness if infection occurs; *(iii)* reducing the probability of clinical symptoms; *(iv)* reducing the average duration of the infectious period. The literature is drawn upon to model these effects and quantify the antiviral efficacy (*AVE*) as in the following (Longini et al. 2004; Longini et al. 2005): AVE_S represents the AV efficacy for susceptibility to infection and corresponds to the reduction of probability of infection for a susceptible taking AV drugs with respect to a susceptible not under prophylaxis; AVE_D

represents the AV efficacy for symptomatic disease given the infection occurs and corresponds to a reduction of the probability of developing symptoms with respect to a person not under prophylaxis; AVE_I represents the AV efficacy for infectiousness and corresponds to the reduction of the probability of transmission of an infectious individual taking AV drugs. The latter effect is valid for both prophylaxis and treatment and also assumes a reduction by 1 day in the average duration of the infection period.

Prophylaxis is administered to a given fraction c_{AVP} of the susceptible population (Flahault et al. 2006; Kernéis et al. 2008), who enter the S_{AVP} compartment and receive a single course of AV for a total duration of 10 days. At the end of this period, they re-enter the susceptible class S in case infection did not occur, since the risk of infection is expected to resume shortly after cessation unless combined with immunization, as for seasonal prophylaxis (World Health Organization 2004b). The parameter c_{AVP} represents the daily coverage of antiviral prophylaxis and is assumed to be equal in all census basins in the world (Flahault et al. 2006; Kernéis et al. 2008). If susceptibles contract the infection while under prophylaxis, they enter the latent class L_{AVP}, which is the analogue of the compartment L and has the same average duration ε^{-1}. Their probability of developing clinical symptoms, $(1-p_a)$ is reduced by a factor $(1-AVE_D)$ as effect of the prophylaxis. Therefore, after an average duration of ε^{-1} days in the L_{AVP} class, the individual either shows influenza symptoms with probability $(1-p_a)(1-AVE_D)$, or becomes asymptomatic and enters the I^a_{AVP} compartment with probability $\left[1-(1-p_a)(1-AVE_D)\right]$. In the case of symptoms, the individual is assumed to be immediately treated, with no further delay, entering the I_{AVT} compartment and starting another course of AV drugs by doubling the dose per day with respect to the prophylaxis. Both I^a_{AVP} and I_{AVT} compartments are characterized by a reduced infectiousness of a factor $(1-AVE_I)$ due to the AV drugs, and by an average duration shortened by 1 day with respect

to an infectious individual not taking drugs, i.e. $\mu_{AV}^{-1} = \mu^{-1} - 1$. We assume that all individuals who get infected while under prophylaxis have access to medical care and get treated with antivirals. In absence of antivirals, they follow the same dynamics as the non-treated infectious individuals (see dashed arrows in Supplemental Figure 1).

Therapeutic treatment is modeled by assuming that: (*i*) all symptomatic infectious individuals under prophylaxis will receive treatment entering the compartment I_{AVT} immediately after the onset of symptoms; (*ii*) symptomatic infectious individuals not under prophylaxis will enter the compartment I_{AVTd} with probability p_{AVT}, a measure of the prompt case detection and rapid drug delivery. The I_{AVTd} compartment includes both the infectious period before the drug administration and the one during the treatment, for a total average duration of $\mu_{AV}^{-1} = \mu^{-1} - 1 = 2d$, with a reduction by 1 day with respect to the mean infectious period with no intervention. We assume that the drugs are administered with a delay of 1 day from the symptoms onset, and that the individual has an effective reduced transmissibility r_{AVTd} that takes into account the pre-treatment period with full infectiousness and the treatment period with reduced infectiousness $\beta(1 - AVE_I)$, i.e., $r_{AVTd}\,\beta = [\beta + \beta(1 - AVE_I)]/2 = [\beta(2 - AVE_I)]/2$.

Influenza-associated pneumonia. Additional compartments and transition rates were introduced to take into account the development of influenza-associated pneumonia (Balcan et al. 2009b). Symptomatic infectious individuals have a certain probability p_P of developing influenza-associated community acquired pneumonia (IA-CAP). The incidence frequency of IA-CAP varies from 2% to 40%, and is dependent on viral and host factors (CM Officer 2005; Lim 2007). The planning assumption of the U.K. Department of Health representing a

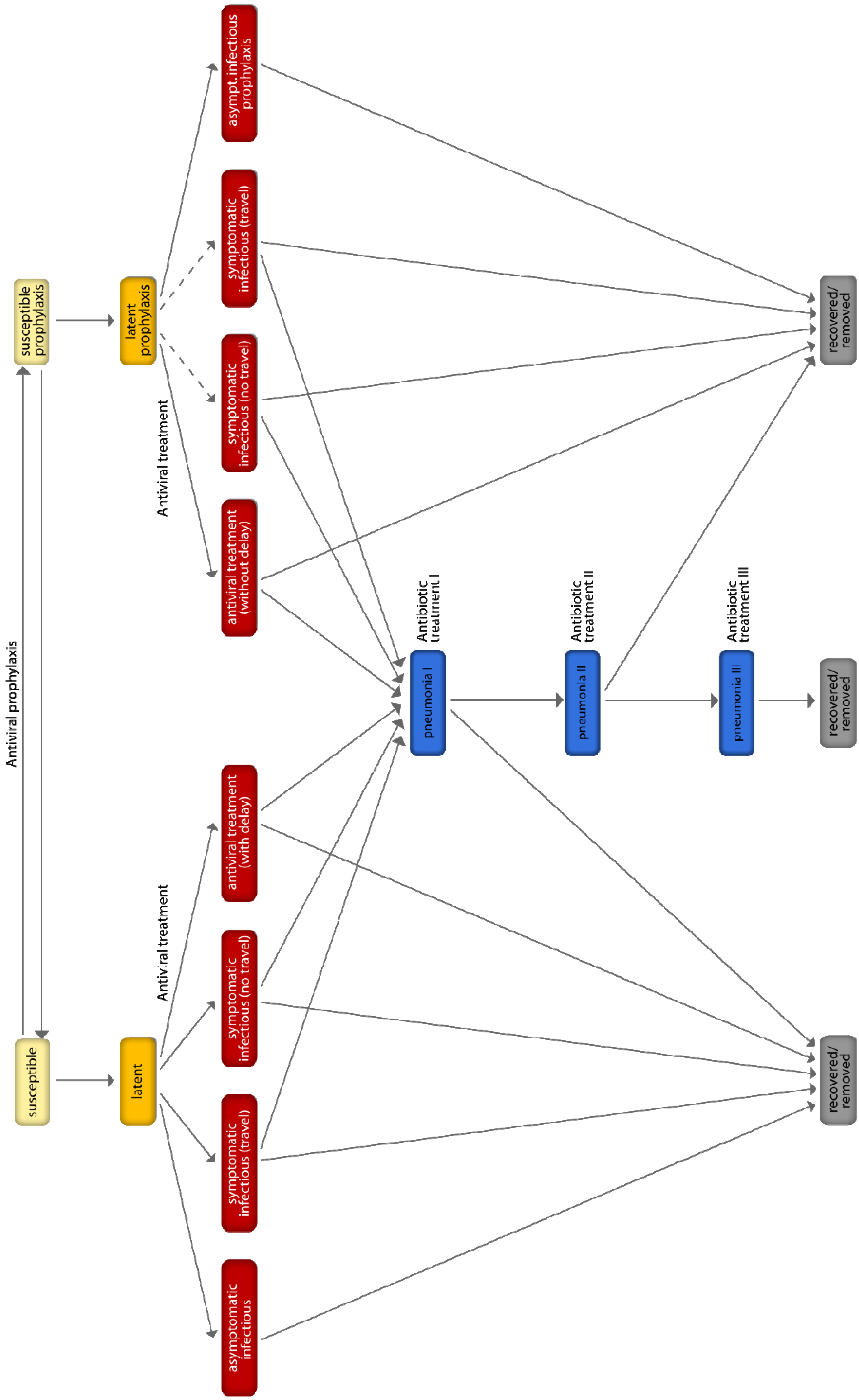

Supplemental Figure 2. Diagram flow of the transmission model.

"reasonable worst case" as of August 31st, 2009, held that 15% of clinical cases will develop complications (U.K. Department of Health 2009b); IA-CAP would be the predominant complication, based on the literature. Here we consider that ill individuals taking antivirals can develop bacterial pneumonia (Brundage and Shanks 2008; Gupta et al. 2008; McCullers and English 2008; Morens et al. 2008; U.K. Department of Health 2007).

Based on recent studies on the impact of oseltamivir treatment on influenza-related lower respiratory tract complications for seasonal flu (Kaiser et al. 2003; Yu et al. 2010), we assume that under AV treatment the probability of developing pneumonia is reduced by a factor α, i.e. $p_{p\text{-}AV}=\alpha\, p_p$, with α=54%. We also assume that all pneumonia cases are treated with antibiotics.

Supplemental Table 2. Severity assessment used to determine the management of influenza-related pneumonia in patients admitted to hospital (CURB-65 score), and corresponding compartments.

**CURB-65 score –** Assign 1 point for each feature present:		
**C**onfusion (mental test score of ≤8, or new disorientation in person, place or time)		
**U**rea >7 mmol/l		
**R**espiratory rate ≥30/min		
**CURB-65**	**Recommended action**	**Compartment**
0-1	non-severe pneumonia, suitable	P^I
2	for home treatment	P^{II}
3-5	increased risk of death, hospital	P^{III}

Multiple subsequent stages of pneumonia course are modelled according to the CURB-65 classification score (Supplemental Table 2). It is assumed that a person will start showing symptoms of IA-CAP after the infectious period is over, entering the compartment P^I, as pneumonia symptoms typically appear during the early convalescent period of the influenza infection (Lim 2007). This corresponds to the stage CURB-65=0 and 1, i.e., non-severe

pneumonia and outpatient care. After an average period of $\lambda^{-1} = 5$ days, p_{II} = 11% of the patients in P^{I} are estimated to experience worsening of symptoms (U.S. Department of Human and Health Services (HHS) 2005), thus requiring hospitalization and entering the compartment P^{II} corresponding to CURB-65 = 2, whereas the rest will recover (with probability 1-p_{II}). Unimproved patients (p_{III} = 1.65%) will develop severe pneumonia and progress to P^{III} corresponding to CURB-65 = 3 to 5. All three stages of pneumonia course (P^{I}, P^{II}, and P^{III}) have an average duration of $\lambda^{-1} = 5$ days (Lim 2007; U.S. Department of Human and Health Services (HHS) 2005). The diagram flow of the epidemic dynamics is shown in Supplemental Figure 2, all parameters values and definition are summarized in Tables S1 and S2 and the predicted usage pattern of antiviral drugs and antibiotics per day in the three transmission scenarios are presented in Supplemental Figure 3.

Supplemental Figure 3. Predicted usage pattern of antiviral drugs and antibiotics per day, respectively, in the three transmission scenarios investigated. The colored solid lines – blue, red, and black – correspond to the predicted median values obtained for the explored values of the reproductive number – R0=1.65, R0=1.9, R0=2.3, respectively. The corresponding shaded areas represent the 95% reference range (RR) emerging from the stochasticity of the processes under study. The time axis indicates the number of days since the start of the pandemic in Southeast Asia. Estimates assume no antiviral prophylaxis in any scenario, no antiviral treatment in the mild transmission scenario, and antiviral treatment of 30% of cases in the moderate and severe transmission scenarios (R0=1.9, R0=2.3). A single course of antibiotics is defined as the combination of antimicrobial drugs considered in the treatment regimen for the suggested duration, following the UK guidelines (Lim 2007).

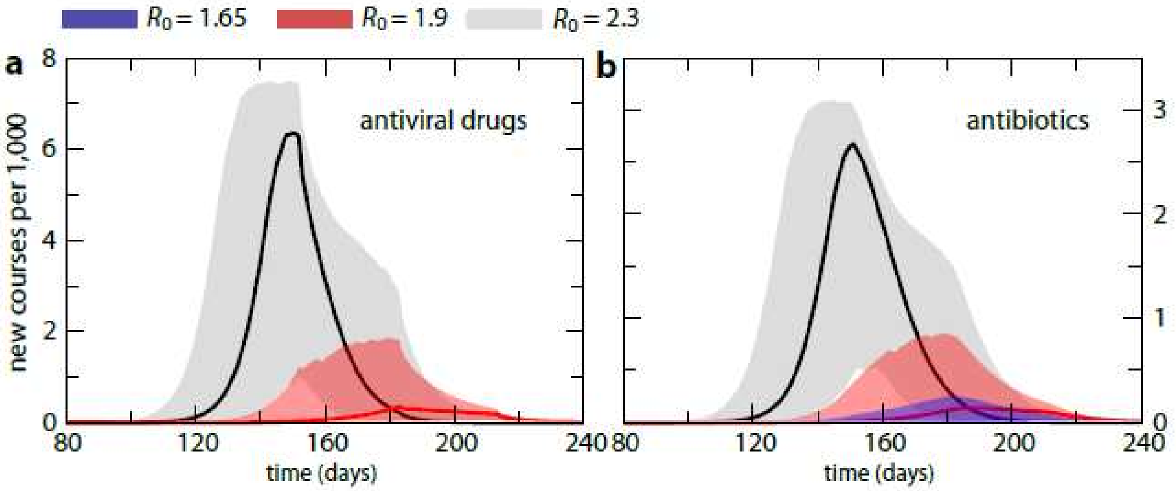

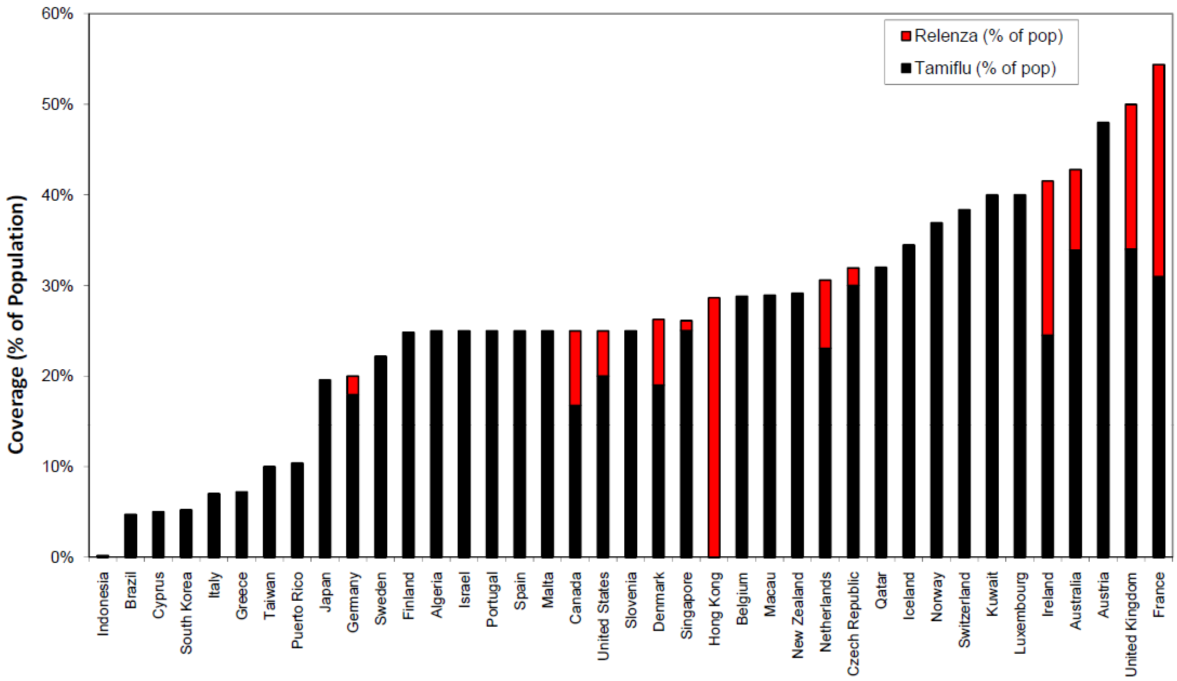

Supplemental Figure 4. Tamiflu (black bar) and Relenza (red bar) stockpiles in national pandemic plans expressed in % of population coverage. This is not an exhaustive list, but has been adapted from the literature (Tierney and Reddy 2007) and media reports and should be used only as a guide. In this paper the antiviral use was assumed to be composed of 100% Tamiflu owing to uncertainties regarding the conditions for Relenza use.

1.2 Pharmaceutical model

Approved antivirals for therapy and prophylaxis of influenza are the neuraminidase inhibitors, i.e., oseltamivir ethylester phosphate (Tamiflu) and zanamivir (Relenza). Relenza stockpiles are increasing due to concerns of Tamiflu resistance (Influenza Project Team 2008; Lackenby et al. 2008; Meijer et al. 2007), however, Tamiflu is currently much more widely stockpiled globally due to the easier administration of the drug (Supplemental Figure 2). Here we consider the use of Tamiflu for therapy and prophylaxis. The consideration of a combination therapy to decrease the risk of developing drug resistance would require a more sophisticated epidemic model that takes into account multiple strains of the virus, and would be considered as a further development of this approach. Individuals under antiviral prophylaxis are given a single course of Tamiflu for 10 days, 1 tablet per day, whereas AVT employs 2 tablets per day for a total duration of 5 days.

During pneumonia stages, patients are given antibiotics, according to the chemotherapy guidelines for treating IA-CAP sanctioned by the British Infection Society, British Thoracic Society and the Health Protection Agency (Lim 2007). It is assumed that empirical antibiotic therapy will be used to treat secondary infections as a result of the overwhelming surge in patients during a pandemic (Lim 2007). In contrast, inter-pandemic IA-CAP treatment would often include laboratory tests to increase the efficiency of the treatment and decrease the likelihood of generating antibiotic resistance. Guidelines recommend the use of 7 days of appropriate antibiotics for patients with non-severe and uncomplicated pneumonia (i.e. compartments P^I and P^{II}), whereas a 10 days treatment is proposed for those with severe pneumonia (P^{III}). Supplemental Table 3 reports the detailed recommendations for the empirical antibiotic treatment regimes.

1.3 Scenarios

All epidemic simulations were initiated with a single symptomatic infectious individual and let evolve for a duration of 1 year. Only the runs with a global outbreak, defined as the generation of new symptomatic cases in more than one country, were considered for the analysis. Initial conditions assume that the pandemic starts in Hanoi on the first of October (Colizza et al. 2007). Simulations with three different values of the reproductive number, R_0=1.65, 1.9, 2.3 have been carried out.

Simulations consider an unlimited stockpile of antibiotics in the UK. In this study, it was assumed that the U.K. will ultimately achieve an antibiotic stockpile sufficiently large to treat

all patients as detailed in the guidelines (Lim 2007), which in some cases would mean a further increase in the stockpile (U.K. Department of Health 2009a).

Supplemental Table 3. Preferred and alternative empirical antibiotic treatment regimens for pneumonic influenza-associated complications (Lim 2007).

CURB-65 score	Compartment	Preferred treatment regimen	Alternative treatment regimen	Duration
**0-2**	P^{I}, P^{II}	co-amoxiclav 625 mg tds PO<br>***or***<br>doxycycline 200mg stat and 100mg od PO	Macrolide<br>(erythromycin 500mg qds PO ***or*** clarithromycin 500mg bd PO)<br>***or***<br>Fluoroquinolone<br>(e.g. levofloxacin 500 mg od PO or moxifloxacin 400 mg od PO)	7 days
**3-5**	P^{III}	co-amoxiclav 1.2g tds IV<br>***or***<br>cefuroxime 1.5g tds IV<br>***or***<br>cefotaxime 1g tds IV<br>***plus***<br>Macrolide<br>(erythromycin 500mg qds IV ***or*** clarithromycin 500mg bd IV)	Fluoroquinolone with some enhanced pneumococcal activity (e.g. levofloxacin 500 mg od IV or moxifloxacin 400 mg od PO)<br>***plus*, either**<br>Macrolide<br>(erythromycin 500mg qds IV ***or*** clarithromycin 500mg bd IV)<br>***or*** Beta-lactamase stable antibiotic (i.e., co-amoxiclav 1.2g tds IV ***or*** cefuroxime 1.5g tds IV ***or*** cefotaxime 1g tds IV)	10 days

2.0 Fate of Pharmaceuticals in the Environment

2.1 Excretion and Dilution of Pharmaceuticals in WWTP

Drug excretion was determined from the pharmacological data within the literature (Dollery 1999; Wishart et al. 2006) as the percentage of drug released in the faeces and urine as the parent chemical, or biologically active metabolite (Supplemental Table 4). The dilution of drug released into the WWTP per day was evaluated using: 1) the mean of all WWTP dry

weather flows (m^3/d) within the Thames river basin as calculated from LF2000-WQX (230 L/head/d; see Supplemental Material Section 2.2); and 2) the actual dry weather flow for each WWTP as found within LF2k-WQX (Supplemental Figure 3). As the per capita consumption of water per day does not vary by more than a factor of 2-3 between industrialised countries (Supplemental Figure 4)(OFWAT 2007), the results of this study might also be applicable to regions that exhibit a similar influenza infection rate and have the capacity to respond with a similar chemotherapy plan.

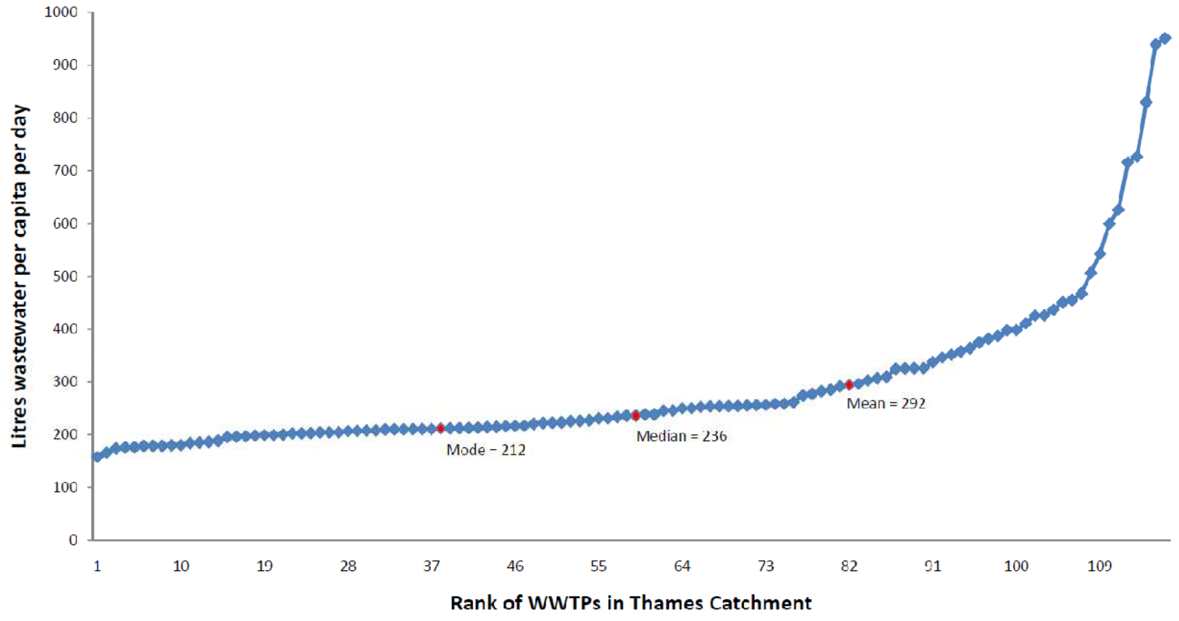

Supplemental Figure 5. Annual mean wastewater produced per head per day (L) in the Thames Catchment as per WWTP database in LF2000-WQX.

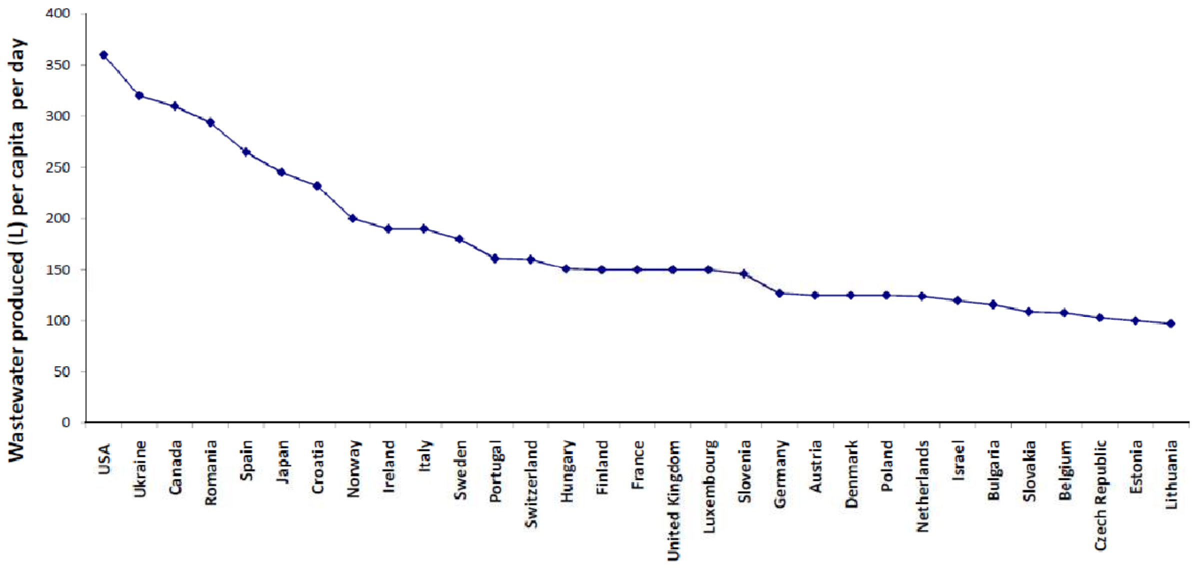

Supplemental Figure 6. International perspective on litres of wastewater production capita^{-1} day^{-1}.

Supplemental Table 4. Percentage of parent pharmaceuticals investigated in this study excreted in the faeces and urine unchanged and/or as a bioactive metabolite.

Pharmaceutical	% Excreted as Parent or Bioactive Metabolite
Tamiflu	100
Amoxicillin	75
Clavulanate	38
Doxycycline	80
Cefuroxime	95
Cefotaxime	61
Erythromycin	100
Clarithromycin	55
Levofloxacin	96
Moxifloxacin	100

Pharmaceuticals in WWTP and rivers. A survey of the literature as well as an examination of the STPWIN model (U.S. Environmental Protection Agency 2007) within the Estimation Program Interface (EPI) Suite^{TM} 4.0, indicates low (<20%) removal for most antibiotics in WWTP, inclusive of loss due to sorption and biodegradation (Supplemental Figure 5). A literature search revealed that most antibiotics, particularly those not containing a β-lactam

moiety, are resistant to metabolism in vivo as well as in the environment (Al-Ahmad et al. 1999; Alexy et al. 2004; Benotti and Brownawell 2009; Brain et al. 2004; Gartiser et al. 2007; Junker et al. 2006; Kümmerer et al. 2000), with half-lives of days to weeks (Benotti and Brownawell 2009; Christensen 1998). It is for these reasons that we feel there is justification in assuming a conservative pharmaceutical biodegradation model of zero degradation/sorption.

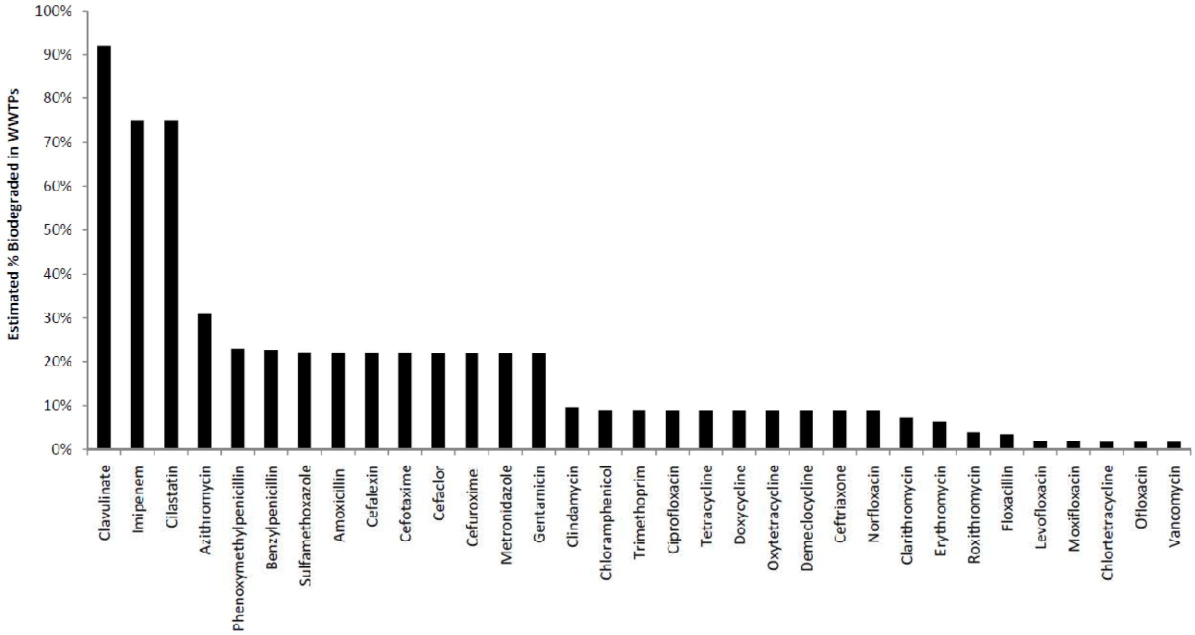

Supplemental Figure 7. Percent removal of a range of antibiotics in WWTP from biodegradation. Loss was estimated using the STPWIN model within the Estimation Program Interface (EPI) Suite^{TM} 4.0, using the Biowin/EPA draft method for determining half-life data (U.S. Environmental Protection Agency 2007).

Photolysis is a major contributor to the degradation of otherwise persistent chemicals in the environment (Andreozzi et al. 2003; Lam et al. 2004). However, laboratory estimates on photodegradability can be misleading as such photo-sensitive chemicals might adsorb into the sediment and organic matter where they remain protected from UV and biological degradation (Andreozzi et al. 2003; Burhenne et al. 1997; Kümmerer and Henninger 2003). As a result of this uncertainty, we have chosen not to include photolysis in calculating

environmental concentrations of pharmaceuticals, conservatively assuming that the antibiotics persist in the environment for more than 1 day.

Tamiflu and oseltamivir carboxylate (OC), the active antiviral of the prodrug Tamiflu, are not anticipated to have any significant biodegradative, sorptive or photoxidative loss within the timeframe of our model (<48 h). Caraciollo et al. (2010) recently showed a significant decline in live cell abundance 14-days after spiking freshwater with OC (Barra Caracciolo et al. 2010). The authors also reported loss of 65% of OC during the 35 d incubation. It is unclear how rapidly this degradation had occurred, as previous timepoints were not provided. Prasse et al. (2010) reported 59% loss of OC within 5 – 12 h (hydraulic retention time) in two full-scale WWTPs (notably the parent chemical oseltamivir was fully-conserved in the same WWTPs)(Prasse et al. 2010). These reports contrast with Accinelli et al. (2010), which recorded 12.8 and 21.0% OC mineralisation in two Japanese river samples, after 40-d incubations (Accinelli et al. 2010). The same study found 75% and <37% OC mineralisation in activated sludge and WWTP effluent-spiked samples over a 40-d period. Bartels and von Tümpling, Jr. (2008) showed an intermediate loss of OC in spiked river water of 50% within 17.8 d (Bartels and von Tümpling Jr 2008). At the other extreme, Slater et al. (In press) and Fick et al. (2007) reported negligible loss of OC during the running of pilot-scale WWTPs (Slater et al. In press; Fick et al. 2007). The variations in OC degradation rates in the literature highlight the difficulty in projecting, with a high degree of certainty, the ecotoxicity of a pandemic-derived bolus of pharmaceuticals on WWTPs and the environment. For this reason a realistic worst case scenario was chosen for populating our chemical fate model in this study, which assumed no loss to sorption, degradation or photolysis, and that all pharmaceuticals would pass through the WWTP into the receiving river in which it will persist for more than 1 day.

2.2 Low Flows 2000 – Water Quality Extension (LF2k-WQX)

The Low Flows 2000 (LF2000) WQX (Water Quality eXtension) model is an extension to the LF2000 system (Young et al. 2003). LF2000 is a decision support tool designed to estimate river flow at gauged and ungauged sites and to assist regional water resources and catchment management.

The LF2000-WQX software (Keller and Young 2004; Williams et al. 2009) is a geographical information-based system that combines hydrological models with a range of water-quality models, including a catchment-scale water-quality model. This model generates spatially explicit statistical distributions of down-the-drain chemicals for both conservative and degradable compounds. It uses a Monte Carlo mixing-model approach to combine statistical estimates of chemical loads at specific emission points (e.g. WWTP) with estimated river flow duration curves for the whole river network of interconnected model reaches (a reach is the river stretch between model features, e.g., major tributaries, sewage treatment works). Thus working from the low order streams at the head of the river network to the outlet of the river basin, the model accounts for the accumulation of point loads and the accumulation of water in which these loads are diluted. Degradable chemicals may be removed from the river water by a non-specific dissipation process assuming first-order kinetics. Details of the model are given in two recent applications: (1) to assess the risk posed to fish by excretion of steroid oestrogens to rivers via sewage treatment works in England and Wales (Williams et al. 2009), and (2) an initial assessment of the likelihood of cytotoxic drugs reaching drinking water in the River Thames basin in south east England (Rowney et al. 2009).

In this study the model was applied to predict the concentrations of Tamiflu and nine antibiotics in the Thames river basin based on per capita excretion of these drugs by the

population. Pharmaceutical load in the WWTP influent per head per day was assumed constant and fixed at the mean peak value for the pandemic. Removal rates in the WWTP were set to zero and the drugs were considered to be stable in the water column (i.e., they were not degraded). The WWTPs were assumed to be the only source of the drugs within the catchment and the background concentrations in the river stretches modelled and lateral inflows to these river stretches was set to zero. The variations of all the model parameters that were defined by a mean and standard deviation were assumed to be described by a normal distribution. Each Monte Carlo simulation comprised 1,350 model runs (shots). For each modelled river reach data were saved in a spatially referenced data file (ArcMap shapefile), which amongst other information, contained the predicted mean concentrations, the standard deviation and the 90th percentile concentration (the concentration which would be predicted not to be exceeded for 90 percent of the time) for each of the modelled drugs.

2.3 Background level of pharmaceuticals in the Thames River

A very wide range of pharmaceuticals are in constant use in a population and thus will be present in the wastewater during a pandemic (Kümmerer 2009a, b). If one was to use the average annual antibiotic usage within England during an inter-pandemic period (NHS BSA 2008) as a first approximation, 62 µg antibiotics/L would be present in the UK WWTPs, which was assumed to be diluted by the median volume of wastewater in WWTP within the Thames Catchment, 230 L/head/day (Supplemental Table 5).

Supplemental Table 5. Estimated concentration of antibiotics in English WWTP during an inter-pandemic period. Drug use was as reported by the National Health Service Business Services Authority (NHS BSA 2008). Where the ADQ (average daily quantity) was unknown, the DDD (defined daily dose)(World Health Organization 2004a) was used to calculate the mass of drug used per head per day (population of England served by the NHS: 54,180,000).

	Combined µg/head/d	% of total antibiotics in use (mass basis)	Estimated concentration in WWTP (µg/L)
Floxacillin + co-Fluampicil	4068	28.0	17.5
Amoxicillin + co-Amoxiclav	3698	25.4	15.9
Cefalexin	2023	13.9	8.68
Erythromycin	1391	9.6	5.97
Ampicillin	840	5.8	3.61
Ciprofloxacin	582	4.0	2.50
Penicillin V	531	3.6	2.28
Trimethoprim	387	2.7	1.66
Cefradine	253	1.7	1.09
Clarithromycin	156	1.1	0.671
Cefaclor	129	0.9	0.553
Cefadroxil	126	0.9	0.543
Clavulanate	108	0.7	0.464
Oxytetracycline	66	0.5	0.285
Lymecycline	47	0.3	0.202
Sulfamethoxazole	36	0.3	0.156
Cefuroxime	24	0.2	0.103
Minocycline	30	0.2	0.128
Doxycycline	12	0.1	0.052
Azithromycin	16	0.1	0.069
Ofloxacin	9	0.1	0.041
Norfloxacin	8	0.1	0.035
Levofloxacin	7	0.1	0.031
Moxifloxacin	5	<0.1	0.022

3.0 Integration of GLEaM with LF2000-WQX

GLEaM and LF-2000-WQX were integrated to study the Thames catchment. All WWTPs in the catchment were mapped into the geographical census areas of GLEaM. The mapping allows estimation of the portion of the population living in the Thames river catchment for each of the geographical census areas, as based on the demographic database at the cell resolution (¼° x ¼°). This preserves the granularity and population heterogeneity in the Thames catchment, in order to calculate the drug usage per person per WWTP.

For each epidemic scenario, characterized by a given transmission scenario and by a given protocol for pharmaceutical interventions, GLEaM allows the simulation, as a function of time, of the number of individuals (*i*) under AV treatment, (*ii*) under AV prophylaxis, (*iii*) under antibiotic treatment in each of the CURB-65 classes. These simulation results enable calculation of the amount of drugs used per person on a daily basis, for each of the drugs considered in the pharmaceutical model based on recommended dosages. Median drug usage values with the corresponding confidence intervals populated the LF2000-WQX model to calculate the concentration of drugs in the Thames catchment. Since LF2000-WQX is not a dynamic model and thus acquires as an input a single value of drug usage in µg/head/day, we considered the median and confidence interval of the peak drug usage rate to assess the highest toxicity in the rivers, given the scenario under study. The day of peak drug usage was used as a worst case scenario for predicted river water concentrations.

4.0 Toxicity Assessment

We focus our ecotoxicologic analysis on antibiotics, as Tamiflu itself has not been shown to exhibit accute toxicity (Accinelli et al. 2010; Bartels and von Tümpling Jr 2008; Hutchinson et al. 2009; Kelleher and Dempsey 2007; Saccà et al. 2009; Straub 2009). Antibiotic sensitivity data for WWTP consortia of sufficient quality is lacking in the literature, as existing data does not allow for the creation of dose-response curves for all antibiotics for the same endpoints. Effects assessment for microorganisms in the WWTP were thus based on the minimum inhibitory concentrations (MICs) of predominantly clinically-relevant microorganisms as a surrogate, as they are among the few standardised measures of microbial inhibition and as environmental bacteria were shown to have similar sensitivities. For a discussion and justification of the use of pathogen MICs, see Section 4.5.

The MIC distributions were from the EUCAST (European Committee on Antimicrobial Susceptibility Testing) breakpoint database (EUCAST 2009), which is a compilation of national and international resistance surveillance programmes, and MIC distributions from published articles, the pharmaceutical industry, veterinary programmes and individual laboratories. The possible synergistic action of clavulanic acid was also not taken into account, as MIC values for the mixture of amoxicillin and clavulanic acid are only published for a mixture of one given composition, while the ratio of clavulanic acid to amoxicillin in the environment will differ from that composition. This will lead to an underestimation of the overall toxicity, however clavulanic acid was included in calculating the values found in Table 1 in the main text. Thus, the effects of 8 antibiotics were studied (amoxicillin, cefotaxime, cefuroxime, clarithromycin, doxycycline, erythromycin, levofloxacin and moxifloxacin).

All MIC values for one given antibiotic were first aggregated into species sensitivity distributions (SSD)(Newman et al. 2002). After calculating the effects of the single antibiotics, the combined effects of the mixture of antibiotics present in STPs and rivers were calculated on the basis of two mixture toxicity models, concentration addition and independent action (De Zwart and Posthuma 2005). All effect calculations were performed in R (R development core team 2009).

4.1 Establishment of species sensitivity distributions

A total of >1,000,000 MIC values were included, including between 21 and 100 bacterial species per antibiotic. SSD curves were constructed to gather the sensitivities of all bacterial species for one given antibiotic in one graph, ordered by their MIC value. The SSD is the cumulative density function giving the “potentially affected fraction” (PAF) of bacteria that are inhibited at a given MIC on a scale between 0 and 1.

For the construction of the SSD curves, three different approaches were applied. The first approach was based on mean MIC values for each species (European Commission 2003). The second, most conservative, approach was based on the 5th percentile of the MIC values of each species. The third approach was based on the distribution of the MIC values per species, termed the 'normalized MIC distribution' (Duboudin et al. 2004). For the first and second approach, the geometric mean and the 5th percentile of the MIC values recorded for each species for each antibiotic were calculated. The aim of the 'normalized MIC distribution' was to retain the intra-species variation of MIC values, since there were numerous MIC values for each species, which showed a wide variation. This was achieved by normalizing the MIC values by the total number of records for this given species, such that each species had the

same total weight in the SSD distribution. This was deemed necessary as the number of MIC values recorded varied between species. The fractions of all bacterial species that were inhibited at one antibiotic concentration were summed up and transformed into an SSD score.

In order to derive dose-response functions of the PAF of bacteria per antibiotic concentration, the SSDs obtained were then fitted by different functions. First, as a pragmatic choice, a lognormal distribution (Aldenberg and Jaworska 2000) was established by the mean and standard deviation of the mean MIC values and 5th percentile MIC values. The suitability of this approach was tested by applying the Anderson-Darling test for normality to the mean and 5th percentile MIC values on the logarithmic scale. For 6 of the 8 studied antibiotics, the mean MIC or 5th percentile MIC values did not pass the Anderson-Darling normality test, and we thus proceeded with applying different distributions.

Second, the SSD distributions were fitted to two-parameter log-logistic and two-parameter Weibull functions (Ritz and Streibig 2005) with package ‘drc’ (Ritz and Streibig 2005). The quality of the curve-fits was judged by their residual variance. In order to compare these residual variances to the log-normal distributions, the residual variation of the SSDs based on the mean MIC values on the log-normal distribution was calculated.

For the SSDs based on the normalized MIC distributions, the asymmetric Weibull function ($f(x) = exp(-exp(b(log(x)-e)))$), with e denoting the logarithm of the inflection point of the sigmoidal curve and x the antibiotic concentration, was the best-fitting function of all three functions for 6 out of the 8 antibiotics, while the log-logistic function was used for the description of the SSD graph of doxycycline and cefuroxime. For the SSDs based on the 5th percentile MICs, doxycycline, cefuroxime, erythromycin and levofloxacin had smaller

residuals with the log-logistic model as compared to the Weibull model. The SSD curves for all antibiotics are given in Supplemental Figure 6.

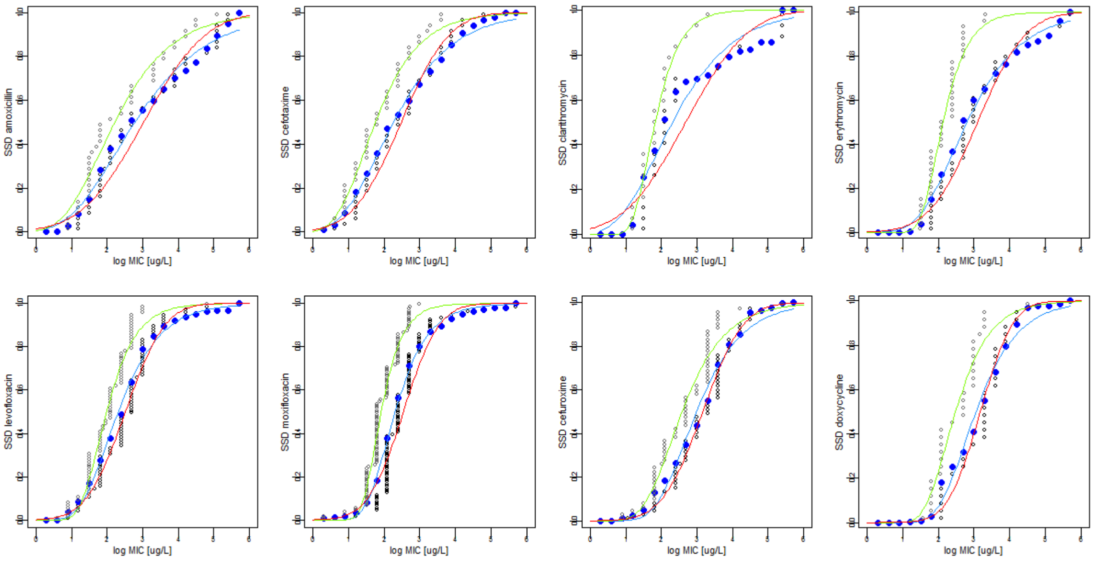

Supplemental Figure 8. Species sensitivity distributions for 8 antibiotics based on MICs acquired from EUCAST database. Black dots: mean MIC values; grey dots: 5-percentile of MIC values; blue dots: SSDs based on normalized MIC distributions. Blue line: Weibull curve fits of normalized MIC distributions, red line: log normal distributions of mean MIC values; green line: Weibull curve fits of 5th percentile of MIC values.

Finally, three different SSD curves were selected:

1. log-normal distribution of the mean MIC values; classic approach, but there are concerns due to non-normality of the MIC distributions
2. best fitting curve (Weibull / logistic) of the 5th percentile MICs; provides a conservative (i.e., worst case) estimate of antibiotic toxicity
3. best fitting curve (Weibull / logistic) of the normalized MIC distributions; represents numerically most accurate approach.

4.2 Calculation of the combined effects of the antibiotic mixture

For the calculation of the multi-substance PAF (msPAF, ie., all antibiotics present simultaneously), two mixture toxicity models were applied to the SSD dose-effect curves

(Altenburger et al. 2000; Backhaus et al. 2000; Bliss and Mexico 1939; De Zwart and Posthuma 2005; Loewe 1927; Loewe and Muischnek 1926). The first model, concentration addition, is generally assumed to hold for compounds with an identical mechanism of action, such as binding to the same receptor. The second, independent action, is assumed to hold for compounds with a different mode of action.

Antibiotics belonging to different classes were assumed to act by independent action (also called dissimilar action), whereas the joint toxicity of antibiotics from identical classes were calculated through concentration addition (similar action). Concentration additivity was thus applied for the pair of fluoroquinolones (moxifloxacin and levofloxacin), both inhibiting gyrase, and the pair of macrolides (erythromycin and clarithromycin), both inhibiting cross-linkage of peptidoglycan chains of the bacterial cell wall. Cefuroxime and cefotaxime were treated as independently acting, as the third-generation cephalosporin cefotaxime has an increased activity towards some enterobacteriaceae as compared to the second-generation cephalosporin, cefuroxime. Finally, amoxicillin and doxycycline were treated as independently acting as they can be assigned to different classes of antibiotics.

First, the joint action of the concentration-additive pairs of antibiotics was calculated. This was done through the toxic unit concept: each WWTP or river concentration of the two antibiotics was divided by the respective mean toxicity of this antibiotic, taken here as the inflection point of the Weibull curve fit (*e*). After summation of the toxic units, the total effect was read off from a Weibull curve with e=1 and the averaged slope of both antibiotics. Second, the joint effect of the two pairs and of the other four antibiotics was calculated from the effect of the single antibiotics (single substance PAF, (ssPAF)) using the concept of independent action:

$$msPAF = 1 - \prod (1 - ssPAF)$$

As all antibiotics finally inhibit bacterial reproduction, which could be seen as common mode of action, and as their joint toxicity might thus fall in between predictions from the two models, calculations were also performed for the assumptions that all antibiotics would act according to concentration addition or independent action (calculations were then based on Weibull curve fits only for the 5th percentile MICs and normalized MIC distributions).

From the possible combinations of the three different SSD curves with the three mixture toxicity models (concentration addition, independent action and the mixed model), we adopted the one with the most refined assumptions: SSD curves based on the complete normalised distribution of MIC values, and a mixed mixture toxicity model with a similar mechanism of action only for antibiotics from the same class. We thus received one toxicity prediction for each location (WWTP/river stretch) and each scenario.

The results were then aggregated by calculating all WWTPs and river stretches that lead to a risk for > 5% of the total bacterial community (potentially-affected fraction: PAF). Five-percent was chosen as a threshold as the definition of predicted no-effect concentrations (PNEC) for industrial chemicals in the European Union, is based on <5% potentially affected fraction of the total species present (based on NOECs, no observed effect concentrations, and taking into account a 50% confidence interval)(European Commission 2003).

4.3 Impact of different toxicity measures

We examined the effect of different microbial toxicity model assumptions on simulated mean toxicity in WWTPs. We compared the influence of changes in both the establishment of the SSD curves and the mixture toxicity assumptions to the chosen, most refined model. Results show that the overall behaviour with respect to the WWTP is left unchanged, with the absolute values of the toxicity varying by about 10-15% (Supplemental Figure 7). We adopted the most refined assumptions: SSD curves based on the complete distribution of MIC values per species, and a mixed mixture toxicity model with a similar mechanism of action only for antibiotics from the same class.

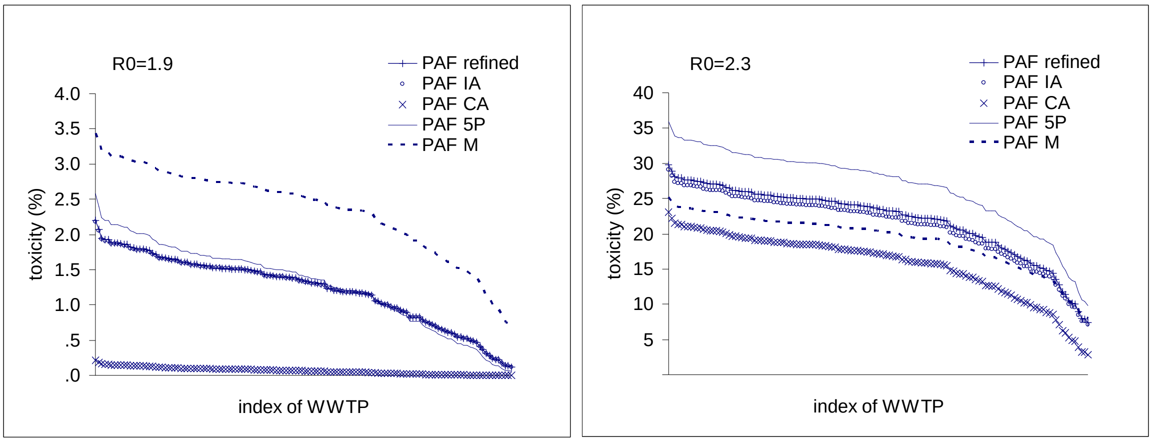

Supplemental Figure 9. Assessment of different toxicity model assumptions as a function of wastewater treatment plants. The scenario considered corresponds to no prophylaxis and AVT=0.3. The influence of mixture toxicity models is shown in PAF IA (all antibiotics independently acting) and PAF CA (all antibiotics acting according to concentration addition), and the influence of the representation of the MIC distributions in the SSD curves in PAF 5P and PAF M (the best fit of the 5th percentile respectively the log-logistic fit of the median of the sensitivity of each bacterial species to one given antibiotic). These are compared to the chosen model PAF refined (based on the normalized distribution of the sensitivity of each species and a combination of both mixture toxicity models, with concentration additivity only assumed for antibiotics from the same class)

4.4 Toxicity of background levels of antibiotics

It was beyond the scope of this study to fully incorporate the toxicity of the pharmaceuticals in constant use (Supplemental Table 5) into the ecotoxicity model. First, pharmaceuticals will not be the only compounds possibly inhibiting bacterial growth, and calculations based on

estimates of antibiotics will underestimate toxicity. Moreover, it is uncertain how usage would shift in light of a pandemic. As a first approximation, the toxicity of the 8 antibiotics used during a pandemic was also calculated for combined pandemic and inter-pandemic use. This will again be an underestimation of the total antibiotic toxicity, as only 8 out of all antibiotics present were taken into account.

The background toxicity of the 8 investigated antibiotics in inter-pandemic times (as in Supplemental Table 5) was calculated to amount to a PAF of 13% for a typical WWTP with a 230 L/head/day dilution (4 to 17% for all WWTP). As the WWTP in the London area are still functioning, an estimated toxicity of 4 to 17% does apparently not cause functional failure. There are several possible reasons: 1) because of community tolerance acquired upon chronic exposure to antibiotics (Blanck 2002); 2) due to other factors that might reduce toxicity, such as sorption and degradation, 3) the main functionally active bacteria have lower sensitivities than these concentrations.

If the concentrations of the 8 antibiotics from inter-pandemic and pandemic use are added up and their toxicity calculated, the lower and the upper RR of WWTP toxicity for the R_0=1.65 scenario range from 3.5 to 13 and 4 to 20% PAF, respectively, reflecting a marginal increase in the background toxicity. For R_0=1.9, the lower RR of all WWTP except 5 would exceed the threshold value of 5% for all possible scenarios. The range of the upper bound of the RR across all different WWTP would increase from 2 to 14% (pandemic use only) to 5 to 25% (inter-pandemic and pandemic antibiotics combined) for the R_0=1.9 scenario. In the R_0=2.3 scenario, maximum toxicities for combined antibiotic use amount from 11% to 37%, compared to 8 to 32 % for pandemic use only. When comparing the median toxicity between the background and the combination of pandemic use and background, the percentage of

growth-inhibited species is elevated by 0.1 to 16% in the latter (median of all WWTPs), with the highest increase for the R_0=2.3 scenario. This is in the same range as the increase in toxicity by pandemic use only (0.03 – 23%).

As the reason for the apparent tolerance of WWTP consortia to background antibiotic concentrations is unknown, it is not possible to precisely estimate how much toxicity is added to the background toxicity by pandemic use. In case the bioavailable concentrations of antibiotics were reduced by e.g. sorption and degradation, one would need exact bioavailability figures to estimate toxicity. There are no toxicological models for an estimation of 'extra' toxicity for the case that the WWTP consortia have become tolerant to inter-pandemic concentrations. Last, if the sensitivity of WWTP consortia is in the upper range of the established SSDs, the likelihood of functional effects increases with increasing percentages of PAF. For these reasons, we decided to focus on the effects of the pandemic antibiotics only. The conclusions will, however, be similar between the approximations above and the analysis presented in the main paper, as both the R0=1.9 and the R0=2.3 scenarios increase overall toxicity by a considerable amount.

4.5 Uncertainties in characterizing toxicity

The models of microbial growth-inhibition resulting from exposure to projected antibiotic concentrations in WWTP during an influenza pandemic have a high degree of uncertainty. Most importantly, the relative sensitivity of WWTP microbial species/consortia to antibiotics, as compared to clinically-relevant human pathogens, remains largely untested (Kümmerer et al. 2000; Robinson et al. 2005). The major difference between clinically-relevant human pathogen MICs and those relevant to WWTPs and the environment, is that environmentally-

relevant MICs would have functional endpoints (i.e., nitrification, mineralization, etc), whereas clinically-relevant endpoints are typically biostasis or death.

The effect of antibiotics on flock-associated WWTP bacteria will undoubtedly differ from effects on planktonic bacteria, as several mechanisms render biofilm consortia more resistant to chemical stress (Donlan and Costerton, 2002). Cells in the centre of the biofilm will be exposed to reduced concentrations of antibiotics due to antibiotic degradation and diffusion limitations. However, the growth rate for the majority of cells was reported to be affected at concentrations of antibiotics comparable to MIC values of planktonic cells, for some antibiotics (Lewis 2000, 2010). Further, the most active cells are present at the flock surface, at least for nitrification (Schramm et al. 1998), where nutrient and antibiotic concentrations are highest. Biofilms might, therefore, be less effective for the protection of WWTP functioning than for the preservation of consortia members located in the centre of biofilms.

Still, a compilation of toxicity data for erythromycin in sewage sludge bacteria allows for a comparison between predictions according to species sensitivity distributions of pathogen MIC values, and experimental data. Experiments were performed with sewage sludge bacterial consortia in batch reactors, and therewith studied relevant microbial consortia under representative conditions. Supplemental Table 6 shows that predictions according to MIC values often lay in between measured effects data for sludge derived from two different WWTP, or were not more than 20% different from the predictions. We thus argue that it is scientifically defensible to use clinically-derived MICs as a first approximation for understanding environmental microbial antibiotic sensitivity.

Supplemental Table 6. Comparison of predicted effects of erythromycin (PAF) with experimentally derived toxicity data.

Ery Conc. [mg/L]	Predicted PAF from Ery exposure [%]	Measured effect from Ery exposure [%]	Toxicity parameter (time of assay)	N	P	Source
**Bactericidal effects**						
0.1	22	10-35	Reduction in live bacteria in mixed liquor samples (20-45 min)	3	NS	1
1	56	50-62	Reduction in live bacteria in mixed liquor samples after (25-45 min)	3	NS	1
**Functional effects**						
1	56	36 / 92 (AS from two different STP)	Batch reactors with AS fed raw wastewater: inhibition of specific NH_4 evolution rate (4 h)	3	NS	2
1	56	51 / 70 (AS from two different STP)	Batch reactors with AS fed raw waste water: Inhibition of the specific COD evolution rate (4h)	3	NS	2
5	75	32	Batch reactors with AS fed raw waste water: Inhibition of the initial ammonia uptake rate over 24 h	Not stated	No	3
10	80	46	Batch reactors with AS fed raw waste water: Inhibition of nitrification (48 h)	Not stated	NS	3
10	80	79 (standard deviation: 34)	Batch reactors with AS fed raw waste water: Inhibition of the specific COD evolution rate (4h)	13	NS	2
10	80	40 (standard deviation: 25)	Batch reactors with AS fed raw waste water: inhibition of specific N-NH_4 evolution rate (4h)	13	NS	2
10	80	38 / -12 (AS from two different STP)	Batch reactors with AS from fed raw waste water: inhibition of the specific nitrification rate	13	NS	2
20	85	66	Batch reactors with AS fed raw waste water: Inhibition of the initial NH_4 uptake rate (24h)	Not stated	Yes	3
20	85	72	Batch reactors with AS fed raw waste water: Inhibition of nitrification (48 h)	Not stated	NS	3

AS = activated sludge, Ery = Erythromycin, PAF = Potentially Effected Fraction; N = number of replicates; P = statistical significance of experimentally measured effect; NS = not stated; Sources: 1- (Louvet et al. In Press); 2- (Louvet et al. 2010); 3- (Alighardashi et al. 2009)

Last, a proper consideration of mixture effects would not only investigate pandemic-derived prescriptions, but a wide range of other inter-pandemic pharmaceuticals (see Supplemental

Table 5), and an even more complex mixture of industrial chemicals and personal care products that are routinely found in wastewater (Alder et al. 2006; Ramirez et al. 2009).

Overall, there is a clear need for empirically-determined thresholds of antibiotic and antiviral toxicity on pilot-scale WWTPs. Significant differences between the microbial consortia indigenous to activated sludge and biofilter systems as well as the heterogeneous distribution of these systems within and between countries (Williams et al. 2009) might require several such pilot-plant studies motivated by different regional or national risks.

4.6 Sensitivity Analysis: Rate of complications development

In the main text we assumed the rate of complications to be equal to 15% of the clinical cases, following the UK Pandemic Assumptions for the current H1N1 influenza pandemic (U.K. Department of Health 2009b). Here we explore different values of the complication rate, ranging from 2% to 40% (CM Officer 2005; Lim 2007). Results are shown in Supplemental Figure 9 and S10 for the toxicity in WWTPs and in rivers, respectively.

When a low complication rate is assumed in a severe scenario, about 45% of the WWTPs would reach a toxicity above the 5% PAF threshold, if AVT=0.3 is assumed. A moderate scenario would not raise toxicity values in WWTPs above the threshold in the interventions considered. The situation is dramatically different in case of a pandemic with a complication rate equal to 40%. Almost the whole set of WWTPs would reach toxicity levels above the threshold in both scenarios R_0=1.9 and R_0=2.3. A mild scenario R0=1.65 would lead 60% to 80% of the WWTPs to reach a toxicity above the 5% PAF threshold. Toxicity higher than the 5% threshold would be observed in a very small fraction of the river stretches, amounting to a negligible fraction of the total river length in the Thames basin, if the complication rate is assumed equal to 2%. When 40% complication rate is considered, about 30 to 40% of the

river stretches would reach a toxicity above threshold in the moderate transmission scenario; the percentage would be twice as much (about 80%) in the case of a severe scenario. When a mild scenario is considered, a very small percentage of the river stretches (about 5 to 10%) would experience toxicity higher than the 5% threshold even in the 40% complication rate assumption.

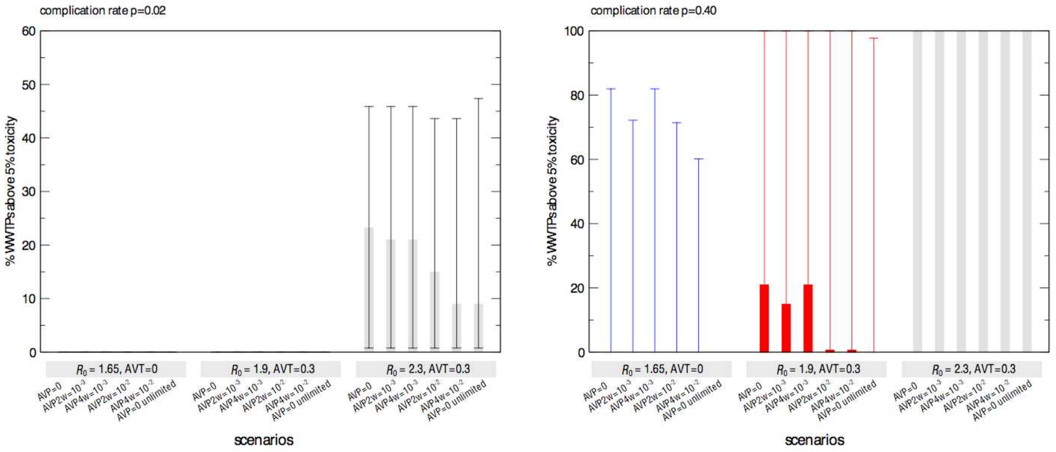

Supplemental Figure 10: Predicted toxicity in WWTPs. Percentage of WWTPs predicted to exceed the threshold of 5% potentially affected species (PAF). Histogram columns and error bars refer, respectively, to the median and 95% confidence intervals obtained from the drugs usage pattern predicted by the 1,000 stochastic runs of the epidemic model. Complication rate equal to 2% (left panel) and 40% (right panel) are shown.

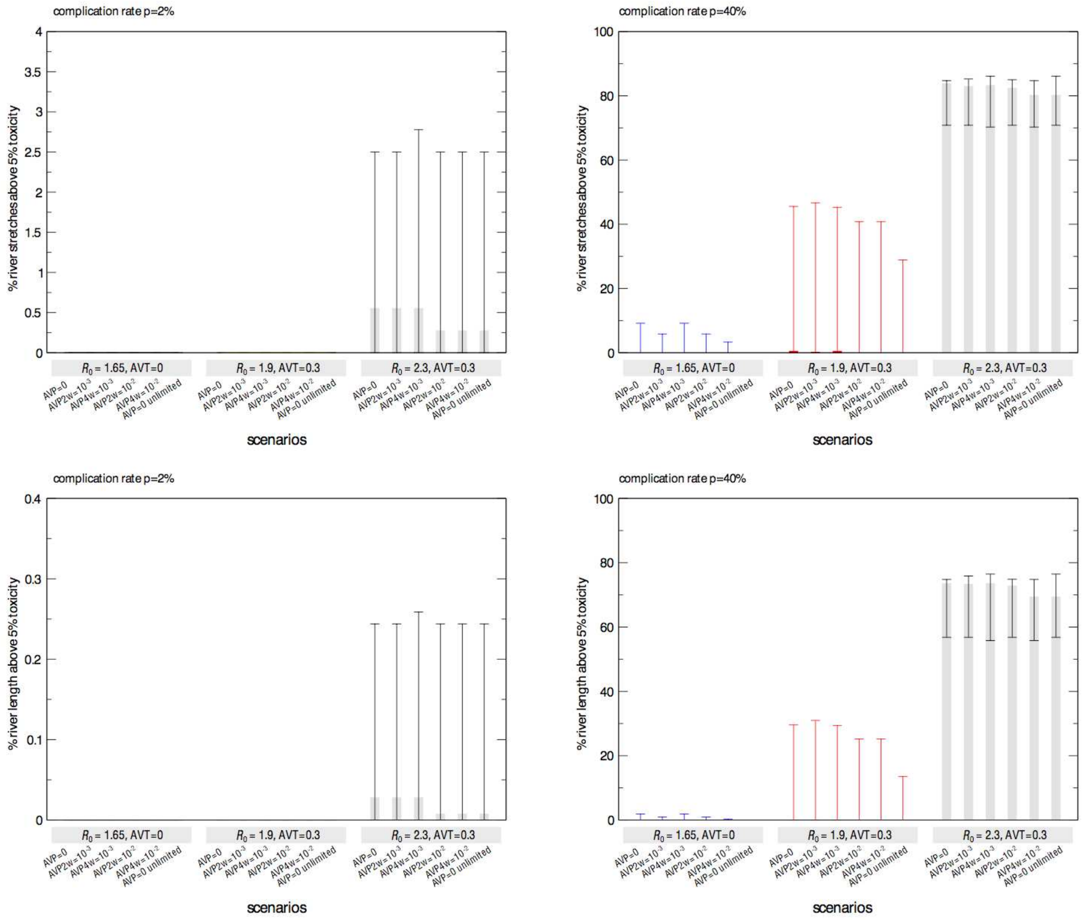

Supplemental Figure 11: Predicted toxicity in river stretches. Percentage of river stretches in the Thames catchment (top row) and percentage of total Thames catchment length (bottom row) predicted to exceed the 5% PAF threshold. Histogram columns and error bars refer, respectively, to the median and 95% confidence intervals obtained from the drugs usage pattern predicted by the 1,000 stochastic runs of the epidemic model. Calculation of the PAF is based on the antibiotic sensitivity distributions of human pathogens and a combination of two mixture toxicity models. Complication rate equal to 2% (left panel) and 40% (right panel) are shown.

5.0 Bacterial Growth and Biofilm Formation Assays

5.1 Bacterial biofilms

Pathogenic bacteria have been shown to produce neuraminidases to assist in the colonization of heavily sialyated secretions and surfaces of the upper respiratory tract (Soong et al. 2006). Limited data exists on the effect of neuraminidase inhibitors on bacterial neuraminidases. Soong et al. (2006) demonstrated the efficiency of oseltamivir carboxylate (active metabolite of Tamiflu) and peramivir to inhibit biofilm formation of the pathogen *Pseudomonas aeruginosa* (Soong et al. 2006). It remains unclear to what extent neuraminidase inhibitors will inhibit biofilm formation in environmentally-relevant strains of microorganisms and how widespread any observed inhibition might be.

Concern has previously been raised regarding the potential for neuraminidase inhibition in bacteria found within WWTP (Singer et al. 2008). The median concentration of Tamiflu in WWTP (assuming 230 L/head wastewater), under a moderate and severe pandemic (AVT=30% and AVP=0) was projected to be approximately 1.00 µg/L and 21.3 µg/L respectively, with maximal values of 5.31 and 102 µg/L OC, respectively. Both scenarios exceed the viral neuraminidase IC_{50} (1.3 nM (0.37 µg/L)(Yamashita et al. 2009), i.e., the concentration at which neuraminidase activity is inhibited by 50%), indicating biologically active concentration of the neuraminidase inhibitors will be present in the WWTP. The concern is that OC might inhibit the capacity for microbial floc and biofilm formation within WWTP. Such inhibition could lead to decreasing wastewater treatment, leading to WWTP failure and contamination of receiving rivers and downstream drinking water abstraction points. Moreover, there is a risk that the effects from neuraminidase inhibitor exposure might be further exaggerated when presented in combination with a high load of antibiotics, as projected in this study.

5.2 Biofilm inhibition assays

Supplemental Table 7. Bacterial strains selected for the biofilm bioassay.

Strain	Origin	Reference
Pseudomonas aeruginosa PAO1	Soil/water/clinical	(Stover et al. 2000)
Pseudomonas fluorescens SBW25	Soil/Phyllosphere	(Rainey and Bailey 1996)
Pseudomonas fluorescens WH2	Groundwater	(Huang et al. 2009)
Acinetobacter sp. Adp1	Soil	(Juni and Janik 1969)
Escherichia coli DH5α™	Clinical	Invitrogen
Pseudomonas putida KT2440 UWC1	Soil	(Bagdasarian et al. 1981)
Rhodococcus sp. RC291	Soil	(Geoghegan et al. 2008)
Pseudomonas Pse1	Groundwater	(Geoghegan et al. 2008)
Bacillus subtilis 168	Soil/water/plants	(Kunst et al. 1997)
Sphingomonas sp. Sph2	Groundwater	(Geoghegan et al. 2008)

A cell attachment assay was used to determine the influence of Tamiflu exposure on biofilm formation of environmentally relevant bacterial strains (Djordjevic et al. 2002; O'Toole and Kolter 1998). One clinical and nine environmental microorganisms were selected from our culture collection (University of Sheffield), representing a range of possible bacteria potentially found within a WWTP and/or the environment (Supplemental Table 7). The isolates were maintained on a solid R2A medium (Oxoid) or for *E. coli* strains on LB agar (Oxoid). Each bacterial strain (Supplemental Table 7) was inoculated into 100 ml of aqueous R2A medium in a 250 ml shake flask with and without one of two nominal concentrations of Tamiflu (oseltamivir), 0.1 and 1.0 mg/L. Tamiflu was acquired from Sequoia Research Products Ltd. (Pangbourne, UK). These cultures were incubated for 24 hr at 30°C in a shaking incubator. Cells were spun down, washed and resuspended in sterile 0.9% NaCl, and the OD_{600} was measured in a spectrophotometer. The cell suspensions were diluted further to produce an OD_{600} of 0.2. A 50 μl aliquot of cell suspension with an OD_{600} of 0.2 was added to 950 μl of R2A in a sterile 1.5 ml micro centrifuge tube, producing a theoretical OD_{600} of

0.01. A 200 µl aliquot of cell suspension was transferred in quadruplicate into sterile 96 well polystyrene plates. Plates were incubated in the dark at 30°C, shaking at 100 rpm. After 96 h the cell cultures were washed using a Beckman Biomek 2000 plate washer to standardise the wash step (Geoghegan et al. 2008), stained with 0.1% crystal violet for 10 minutes before the crystal violet was released using 33% acetic acid. The crystal violet was then removed to a clean 96-well plate and the absorbance was measured at 630 nm.

There was a statistically significant ($p<0.05$) decline in biofilm formation relative to the control in 20% and 10% of the cases when the bacterial isolates were exposed to 0.1 mg/L and 1 mg/L Tamiflu, respectively (Supplemental Figure 12). Notably, one bacterial isolate *Pseudomonas fluorescens* WH2 exhibited a significant increase in biofilm formation suggestive of growth on Tamiflu. Although there was a substantial change in biofilm formation in most isolates, these changes were not significant at the $p<0.05$ level. Additional research would be required: 1) to examine a wider range of Tamiflu and the active antiviral, oseltamivir carboxylate (OC); 2) to examine a more diverse range of microorganisms to determine how widespread the biofilm disruption may be and the mechanism behind such a disruption; 3) to examine mixed cultures, particularly those of a WWTP and river microbial biofilms to address community dynamics; 4) to examine the added effect of chemical mixtures, i.e., antibiotics and Tamiflu; and 5) to examine all available neuraminidase inhibitors, as Tamiflu is only one neuraminidase inhibitor in a growing class of drugs. Varying IC's for OC and peramivir indicate the likelihood that each neuraminidase inhibitors will potentially have a different effect on microbial biofilms and toxicity (Soong et al. 2006).

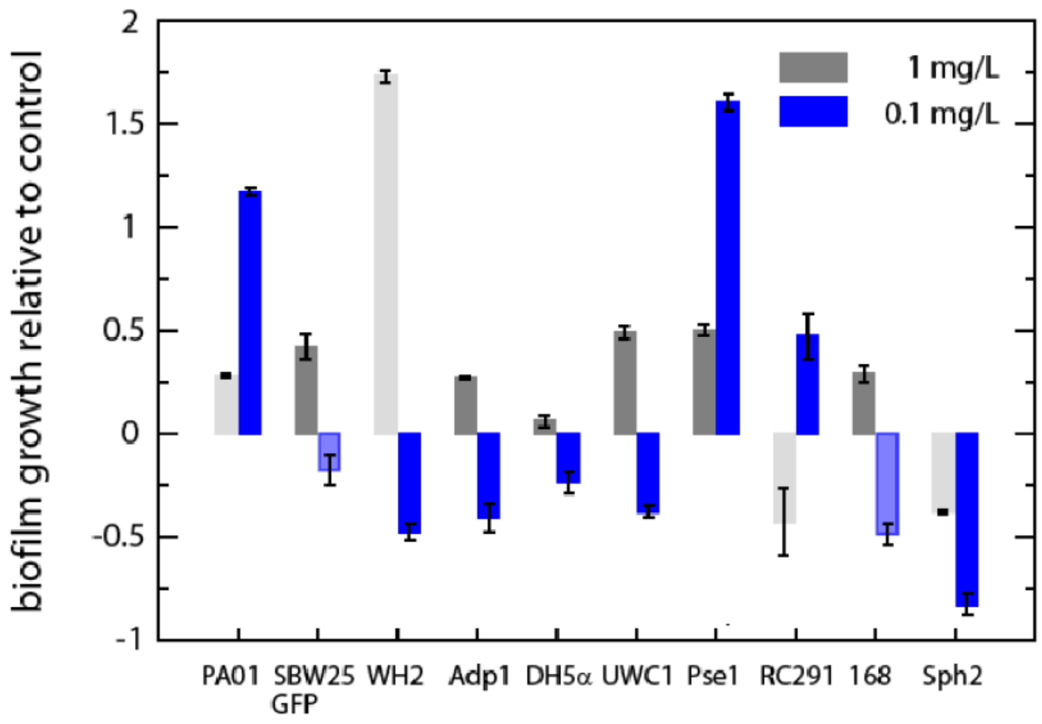

Supplemental Figure 12: Impact of Tamiflu exposure on microbial biofilm formation. Fraction increase or decrease in biofilm formation for a range of ten microorganisms (Supplemental Table 7) in the presence of 1.0 μg L^{-1} (grey bar) or 0.1 μg L^{-1} (blue bar) Tamiflu. Changes are statistically significant where denoted by a lighter shaded grey or blue bar (Students t-test at $p<0.05$). Error bars denote standard error of the mean (n=4).

6.0 References

Accinelli C, Saccà ML, Fick J, Mencarelli M, Lindberg R, Olsen B. 2010. Dissipation and removal of oseltamivir (Tamiflu) in different aquatic environments. Chemosphere 79(8): 891-897.

Al-Ahmad A, Daschner FD, Kümmerer K. 1999. Biodegradability of cefotiam, ciprofloxacin, meropenem, penicillin G, and sulfamethoxazole and inhibition of waste water bacteria. Arch Environ Contam Toxicol 37(2): 158-163.

Aldenberg T, Jaworska JS. 2000. Uncertainty of the hazardous concentration and fraction affected for normal species sensitivity distributions. Ecotox Environ Safe 46: 1-18.

Alder AC, Bruchet A, Carballa M, Clara M, Joss A, Löffler D, et al. 2006. Human pharmaceuticals, hormones and fragrances: the challenge of micropollutants in urban water management. London UK: IWA Publishing.

Alexy R, Kumpel T, Kümmerer K. 2004. Assessment of degradation of 18 antibiotics in the closed bottle test. Chemosphere 57(6): 505-512.

Alighardashi A, Pandolfi D, Potier O, Pons MN. 2009. Acute sensitivity of activated sludge bacteria to erythromycin. J Hazard Mater 172(2-3): 685-692.

Altenburger R, Backhaus T, Boedeker W, Faust M, Scholze M, Grimme LH. 2000. Predictability of the toxicity of multiple chemical mixtures to Vibrio fischeri: Mixtures composed of similarly acting chemicals. Environ Toxicol Chem 19(9): 2341-2347.

Andreozzi R, Raffaele M, Nicklas P. 2003. Pharmaceuticals in STP effluents and their solar photodegradation in aquatic environment. Chemosphere 50(10): 1319-1330.

Backhaus T, Altenburger R, Boedeker W, Faust M, Scholze M, Grimme LH. 2000. Predictability of the toxicity of a multiple mixture of dissimilarly acting chemicals to Vibrio fischeri. Environ Toxicol Chem 19(9): 2348-2356.

Bagdasarian M, Lurz R, Ruckert B, Franklin FCH, Bagdasarian MM, Frey J, et al. 1981. Specific-purpose plasmid cloning vectors. 2. Broad host range, high copy number, RSF1010-derived vectors and a host-vector system for gene cloning in pseudomonas Gene 16(1-3): 237-247.

Balcan D, Colizza V, Gonçalves B, Hu H, Ramasco JJ, Vespignani A. 2009a. Multiscale mobility networks and the large scale spreading of infectious diseases. Proc Natl Acad Sci USA 106: 21484-21489.

Balcan D, Colizza V, Singer AC, Valleron A-J, Chouaid C, Vespignani A. 2009b. Pandemic influenza and bacterial pneumonia: managing antiviral and anitbiotic resources.

Balcan D, Hu H, Goncalves B, Bajardi P, Poletto C, Ramasco J, et al. 2009c. Seasonal transmission potential and activity peaks of the new influenza A(H1N1): a Monte Carlo likelihood analysis based on human mobility. BMC Medicine 7(1): 45.

Barrat A, Pastor-Satorras R, Vespignani A. 2004. The architecture of complex weighted networks. Proc Natl Acad Sci USA 101(3747-3752).

Bartels P, von Tümpling Jr W. 2008. The environmental fate of the antiviral drug oseltamivir carboxylate in different waters. Sci Total Environ 405(1-3): 215-225.

Benotti MJ, Brownawell BJ. 2009. Microbial degradation of pharmaceuticals in estuarine and coastal seawater. Environ Pollut 157(3): 994-1002.

Blanck H. 2002. A critical review of procedures and approaches used for assessing pollution-induced community tolerance (PICT) in biotic communities. Hum Ecol Risk Assess 8(5): 1003-1034.

Bliss CI, Mexico DF. 1939. The toxicity of poisons applied jointly. Ann Appl Biol 26: 585-615.

Brain RA, Johnson DJ, Richards SM, Sanderson H, Sibley PK, Solomon KR. 2004. Effects of 25 pharmaceutical compounds to *Lemna gibba* using a seven-day static-renewal test. Environ Toxicol Chem 23(2): 371-382.

Brundage JF, Shanks GD. 2008. Deaths from Bacterial Pneumonia during 1918–19 Influenza Pandemic. Emerg Infect Dis 14(8): 1193-1198.

Burhenne J, Ludwig M, Nikoloudis P, Spiteller M. 1997. Photolytic degradation of fluoroquinolone carboxylaic acids in aqueous solution. Primary photoproducts and half-lives. Environ Sci Pollut Res 4(10-15).

Center for International Earth Science Information Network (CIESIN) Columbia University, International Food Policy Research Institute (IFPRI), The World Bank, Centro Internacional de Agricultura Tropical (CIAT). Global Rural-Urban Mapping Project (GRUMP), Alpha Version: Population Grids. Palisades, NY: Socioeconomic Data and Applications Center (SEDAC), Columbia University. . Available at: http://sedac.ciesin.columbia.edu/gpw [accessed 30 June 2010].

Christensen FM. 1998. Pharmaceuticals in the environment: a human risk. Regulatory. Toxicology & Pharmacology 28: 212-221.

CM Officer. 2005. CMO Annual Report. Planning for a rising tide: being prepared for the flu pandemic. . Available at: http://www.dh.gov.uk/prod_consum_dh/idcplg?IdcService=GET_FILE&dID=116004&Rendition=Web [accessed 12 Jan 2010].

Colizza V, Barrat A, Barthelemy M, Valleron A-J, Vespignani A. 2007. Modeling the woldwide spread of pandemic influenza: baseline case and containment interventions. PLoS Medicine 4(1): e13.
Colizza V, Barrat A, Barthélemy M, Vespignani A. 2006a. The modeling of global epidemics: stochastic dynamics and predictability. Bull Math Biol 68(8): 1893-1921.
Colizza V, Barrat A, Barthélemy M, Vespignani A. 2006b. The role of the airline transportation network in the prediction and predictability of global epidemics. Proc Natl Acad Sci U S A 103(7): 2015-2020.
De Zwart D, Posthuma L. 2005. Complex mixture toxicity for single and multiple species: Proposed methodologies. Environ Toxicol Chem 24(10): 2665-2676.
Djordjevic D, Wiedmann A, McLandsborough LA. 2002. Microtiter plate assay for assessment of *Listeria monocytogenes* biofilm formation. Appl Environ Microbiol 68(6): 2950-2958.
Dollery C. 1999. Therapeutic Drugs. 2nd ed. London: Harcourt Brace and Company Limited.
Duboudin C, Ciffroy P, Magaud H. 2004. Effects of data manipulation and statistical methods on species sensitivity distributions. Environ Toxicol Chem 23(2): 489-499.
EUCAST. 2009. Antimicrobial wild type distributions of microorganisms. Available at: http://wwweucastorg/clinical_breakpoints/ [accessed 22 February 2011].
European Commission. 2003. Part II, Chapter 3 - Environmental risk assessment. (Technical Guidance Document in support of Commission Directive 93/67/EEC on Risk Assessment for new notified substances, Commission Regulation (EC) No 1488/94 on Risk Assessment for existing substances and Directive 98/8/EC of the European Parliament and of the Council concerning the placing of biocidal products on the market). EUR 20418 EN/2. Ispra:European Commission, Institute for Health and Consumer Protection - European Chemicals Bureau.
Flahault A, Vergu E, Coudeville L, Grais RF. 2006. Strategies for containing a global influenza pandemic. Vaccine 24(44-46): 6751-6755.
Gartiser S, Urich E, Alexy R, Kümmerer K. 2007. Ultimate biodegradation and elimination of antibiotics in inherent tests. Chemosphere 67(3): 604-613.
Geoghegan M, Andrews J, Biggs C, Eboigbodin K, Elliott D, Rolfe S, et al. 2008. The polymer physics and chemistry of microbial cell attachment and adhesion. Faraday Discuss 139: 85-103.
Gupta RK, George R, Nguyen-Van-Tam JS. 2008. Bacterial pneumonia and pandemic influenza planning. Emerg Infect Dis 14(8): 1187-1192.
Huang WE, Ferguson A, Singer AC, Lawson K, Thompson IP, Kalin RM, et al. 2009. Resolving Genetic Functions within Microbial Populations: In Situ Analyses Using rRNA and mRNA Stable Isotope Probing Coupled with Single-Cell Raman-Fluorescence In Situ Hybridization. Applied and Environmental Microbiology 75(1): 234-241.
Hutchinson TH, Beesley A, Frickers PE, Readman JW, Shaw JP, Straub JO. 2009. Extending the environmental risk assessment for oseltamivir (Tamiflu®) under pandemic use conditions to the coastal marine compartment. Environ Int 35(6): 931-936.
Influenza Project Team. 2008. Oseltamivir resistance in human seasonal influenza viruses (A/H1N1) in EU and EFTA countries: an update. Eurosurveillance 13(6): 8032.
International Air Transport Association. Available at: http://www.iata.org [accessed 30 June 2010].
Juni E, Janik A. 1969. Transformation of *Acinetobacter calco-aceticus* (*Bacterium anitratum*). J Bacteriol 98(1): 281-288.

Junker T, Alexy R, Knacker T, Kümmerer K. 2006. Biodegradability of ^{14}C-Labeled Antibiotics in a Modified Laboratory Scale Sewage Treatment Plant at Environmentally Relevant Concentrations. Environ Sci Technol 40(1): 318-324.
Kaiser L, Wat C, Mills T, Mahoney P, Ward P, Hayden F. 2003. Impact of oseltamivir treatment on influenza-related lower respiratory tract complications and hospitalizations. Arch Intern Med 163(14): 1667-1672.
Kelleher SL, Dempsey MJ. 2007. The effect of oseltamivir carboxylate on nitrification of wastewater by bioparticles from a pilot-scale expanded bed reactor (poster). Workshop: Tamiflu and the environment: Implication of use under pandemic conditions October 3, 2007, Magdalen College, Oxford, UK Available at: http://www.scribd.com/doc/33746728/The-effect-of-oseltamivir-carboxylate-on-nitrification-of-wastewater-by-bioparticles-from-a-pilot-scale-expanded-bed-reactor-poster (accessed 22 February 2011).
Keller VD, Young AR. 2004. Development of the integrated water resources and water qualtity modelling system. Science Report P2-248/SR Bristol, UK.
Kernéis S, Grais RF, Boëlle P-Y, Flahault A, Vergu E. 2008. Does the Effectiveness of Control Measures Depend on the Influenza Pandemic Profile? PLoS ONE 3(1): e1478.
Kümmerer K. 2009a. Antibiotics in the aquatic environment - A review - Part I. Chemosphere 75(4): 417-434.
Kümmerer K. 2009b. Antibiotics in the aquatic environment - A review - Part II. Chemosphere 75(4): 435-441.
Kümmerer K, Al-Ahmad A, Mersch-Sundermann V. 2000. Biodegradability of some antibiotics, elimination of the genotoxicity and affection of wastewater bacteria in a simple test. Chemosphere 40(7): 701-710.
Kümmerer K, Henninger A. 2003. Promoting resistance by the emission of antibiotics from hospitals and households into effluent. Clinical Microbiology and Infection 9(12): 1203-1214.
Kunst F, Ogasawara N, Moszer I, Albertini AM, Alloni G, Azevedo V, et al. 1997. The complete genome sequence of the Gram-positive bacterium Bacillus subtilis. Nature 390(6657): 249-256.
Lackenby A, Hungnes O, Dudman SG, Meijer A, Paget WJ, Hay AJ, et al. 2008. Emergence of resistance to oseltamivir among influenza A(H1N1) viruses in Europe. Eurosurveillance 13(5): 8026.
Lam MW, Young CJ, Brain RA, Johnson DJ, Hanson MA, Wilson CJ, et al. 2004. Aquatic persistence of eight pharmaceuticals in a microcosm study. Environ Toxicol Chem 25(6): 1431-1440.
Lewis K. 2000. Programmed death in bacteria. Microbiol Mol Biol Rev 64(3): 503-514.
Lewis K. 2010. Persister cells. Annu Rev Microbiol 64: 357-372.
Lim WS. 2007. Pandemic flu: clinical management of patients with an influenza-like illness during an influenza pandemic. Thorax 62(suppl_1): 1-46.
Loewe S. 1927. Die Mischarznei. Versuch einer allgemeinen Pharmakologie der Arzneikombinationen. Klnische Wochenschrift 6: 1077-1085.
Loewe S, Muischnek H. 1926. Über Kombinationswirkungen, 1. Mitteilung: Hilfsmittel der Fragestellung. Nanyn-Schmiedebergs Archiv für Experimentelle Pathololologische Pharmakologie 114: 313-326.
Longini IM, Jr., Halloran ME, Nizam A, Yang Y. 2004. Containing Pandemic Influenza with Antiviral Agents. Am J Epidemiol 159(7): 623-633.
Longini IM, Jr., Nizam A, Xu S, Ungchusak K, Hanshaoworakul W, Cummings DAT, et al. 2005. Containing Pandemic Influenza at the Source. Science 309(5737): 1083-1087.

Louvet J-N, Heluin Y, Attik G, Dumas D, Potier O, Pons M-N. In Press. Assessment of erythromycin toxicity on activated sludge via batch experiments and microscopic techniques (epifluorescence and CLSM). Process Biochem.
Louvet JN, Giammarino C, Potier O, Pons MN. 2010. Adverse effects of erythromycin on the structure and chemistry of activated sludge. Environmental Pollutution 158(3): 688-693.
McCullers JA, English BK. 2008. Improving therapeutic strategies for secondary bacterial pneumonia following influenza. Future Microbiology 3(4): 397-404.
Meijer A, Lackenby A, Hay A, Zambon M. 2007. Influenza antiviral susceptibility monitoring activities in relation to national antiviral stockpiles in Europe during the winter 2006/2007 season. EuroSurveillance 12(4): 698.
Morens DM, Taubenberger JK, Fauci AS. 2008. Predominant role of bacterial pneumonia as a cause of death in pandemic influenza: implications for pandemic influenza preparedness. The Journal of Infectious Diseases 198(7): 962-970.
Newman MC, Ownby DR, Mezin LCA, Powell DC, Christensen TRL, Lerberg SB, et al. 2002. Species sensitivity distributions in ecological risk assessment: distributional assumptions, alternate bootstrap techniques and estimation of adequate number of species. In: Species sensitivity distributions in ecotoxicology, (Posthuma L, II GWS, Traas TP, eds). Boca Raton:CRC Press, p616.
NHS BSA. 2008. Prescribing Analysis Charts: National Antibiotics Charts. Available at: http://www.nhsbsa.nhs.uk/PrescriptionServices/Documents/NPC_Antibiotics_July_2008.ppt [accessed 30 June 2010].
O'Toole GA, Kolter R. 1998. Initiation of biofilm formation in Pseudomonas fluorescens WCS365 proceeds via multiple, convergent signalling pathways: a genetic analysis. Mol Microbiol 28(3): 449-461.
OFWAT. 2007. International comparison of water and sewerage service 2007 report. Available at: http://wwwofwatgovuk/regulating/reporting/rpt_int2007pdf [accessed 12 Jan 2010].
R development core team. 2009. R: a language and environment for statistical computing-. Vienna, Austria: R foundation for statistical computing.
Rainey PB, Bailey MJ. 1996. Physical and genetic map of the *Pseudomonas fluorescens* SBW25 chromosome. Mol Microbiol 19(3): 521-533.
Ramirez AJ, Brain RA, Usenko S, Mottaleb MA, Donnell JG, Stahl LL, et al. 2009. Occurrence of pharmaceuticals and personal care products (PPCPs) in fish: Results of a national pilot study in the U.S. Environ Toxicol Chem preprint(2009): 0000-0000.
Ritz C, Streibig JC. 2005. Bioassay analysis using R. Journal of Statistical Software 12(5): 1-22.
Robinson AA, Belden JB, Lydy MJ. 2005. Toxicity of fluoroquinolone antibiotics to aquatic organisms. Environ Toxicol Chem 24(2): 423-430.
Rowney NC, Johnson AC, Williams RJ. 2009. Cytotoxic drugs in drinking water: a prediction and risk assessment exercise for the Thames catchment in the United Kingdom. Environ Toxicol Chem 28(12): 2733-2743.
Saccà ML, Accinelli C, Fick J, Lindberg R, Olsen B. 2009. Environmental fate of the antiviral drug Tamiflu in two aquatic ecosystems. Chemosphere 75(1): 28-33.
Schramm A, De Beer D, Wagner M, Amann R. 1998. Identification and activities in situ of Nitrosospira and Nitrospira spp. as dominant populations in a nitrifying fluidized bed reactor. Appl Environ Microbiol 64(9): 3480-3485.
Singer AC, Howard BM, Johnson AC, Knowles CJ, Jackman S, Accinelli C, et al. 2008. Meeting report: risk assessment of Tamiflu use under pandemic conditions. Environ Health Perspect 116(11): 1563-1567.

Soong G, Muir A, Gomez MI, Waks J, Reddy B, Planet P, et al. 2006. Bacterial neuraminidase facilitates mucosal infection by participating in biofilm production. J Clin Invest 116(8): 2297-2305.

Stover CK, Pham XQ, Erwin AL, Mizoguchi SD, Warrener P, Hickey MJ, et al. 2000. Complete genome sequence of Pseudomonas aeruginosa PAO1, an opportunistic pathogen. Nature 406(6799): 959-964.

Straub JO. 2009. An environmental risk assessment for oseltamivir (Tamiflu®) for sewage works and surface waters under seasonal-influenza- and pandemic-use conditions. Ecotox Environ Safe 72: 1625-1634.

Tierney E, Reddy D. 2007. Roche Media Briefing: Update on current developments around Tamiflu (Basel, 26 April 2007). Available at: http://wwwrochecom/media/events/media-briefing-tamiflu-2007htm [accessed 12 Jan 2010].

U.K. Department of Health. 2007. The use of antibiotics for pandemic influenza – Scientific evidence base. Available at: http://www.dh.gov.uk/prod_consum_dh/groups/dh_digitalassets/@dh/@en/documents/digitalasset/dh_077274.pdf [accessed 30 June 2010].

U.K. Department of Health. 2009a. Pandemic flu drug stockpiles are set to double. Available at: http://ndscoigovuk/content/detailaspx?NewsAreaId=2&ReleaseID=391247&SubjectId=16&AdvancedSearch=true [accessed 12 Jan 2010].

U.K. Department of Health. 2009b. Swine Flu: UK planning assumptions. Available at: http://www.dh.gov.uk/en/Publicationsandstatistics/Publications/PublicationsPolicyAndGuidance/DH_104844 [accessed 22 February 2011].

U.S. Department of Human and Health Services (HHS). 2005. Pandemic Influenza Plan. Available at: http://www.hhs.gov/pandemicflu/plan/pdf/HHSPandemicInfluenzaPlan.pdf [accessed 30 June 2010].

U.S. Environmental Protection Agency. 2007. Estimation Program Interface (EPI) Suite v3.2. Available at: http://wwwepagov/oppt/exposure/pubs/episuitehtm [accessed 12 Jan 2010].

Williams RJ, Keller VDJ, Johnson AC, Young AR, Holmes MGR, Wells C, et al. 2009. A national risk assessment for intersex in fish arising from steroid estrogens. Environ Toxicol Chem 28(1): 220-230.

Wishart DS, Knox C, Guo AC, Shrivastava S, Hassanali M, Stothard P, et al. 2006. DrugBank: a comprehensive resource for in silico drug discovery and exploration. Nucl Acids Res 34(suppl_1): D668-672.

World Health Organization. 2004a. The anatomical therapeutic chemical classification system with defined daily doses (ATC/DDD). Norway: WHO: Available: http://www.whocc.no/atcddd/indexdatabase/index.php [accessed 8 July 2008].

World Health Organization. 2004b. WHO guidelines on the use of vaccines and antivirals during influenza pandemics. Available at: http://www.who.int/csr/resources/publications/influenza/11_29_01_A.pdf [accessed 30 June 2010].

Yamashita M, Tomozawa T, Kakuta M, Tokumitsu A, Nasu H, Kubo S. 2009. CS-8958, a Prodrug of the New Neuraminidase Inhibitor R-125489, Shows Long-Acting Anti-Influenza Virus Activity. Antimicrob Agents Chemother 53(1): 186-192.

Young AR, Grew R, Holmes MGR. 2003. Low Flows 2000: a national water resources assessment and decision support tool. Water Sci Technol 48(10): 119-126.

Yu H, Liao Q, Yuan Y, Zhou L, Xiang N, Huai Y, et al. 2010. Effectiveness of oseltamivir on disease progression and viral RNA shedding in patients with mild pandemic 2009 influenza A H1N1: opportunistic retrospective study of medical charts in China. BMJ 341: 341:c4779.
